# Consensus paper on the management of acute isolated vertigo in the emergency department

**DOI:** 10.1007/s11739-024-03664-x

**Published:** 2024-07-13

**Authors:** Simone Vanni, Paolo Vannucchi, Rudi Pecci, Giuseppe Pepe, Maurizio Paciaroni, Andrea Pavellini, Mattia Ronchetti, Lorenzo Pelagatti, Maurizio Bartolucci, Angela Konze, Andrea Castellucci, Marco Manfrin, Andrea Fabbri, Fabio de Iaco, Augusto Pietro Casani, Società Italiana di Medicina d’Emergenza Urgenza [SIMEU], Società Italiana di Vestibologia [VIS]

**Affiliations:** 1 Dipartimento di Medicina Sperimentale e Clinica, Università degli studi di Firenze, Largo Brambilla 3, 50134 Florence, Italy; 2 Medico Audiologo, Florence, Italy; 3 Audiologia, Azienda Ospedaliero Universitaria Careggi, Florence, Italy; 4 Medicina Emergenza Urgenza e Pronto Soccorso, Azienda USL Toscana Nord Ovest, Ospedale Versilia, Viareggio, Italy; 5 Medicina Interna e Cardiovascolare, Stroke Unit, Santa Maria della Misericordia Hospital, Perugia, Italy; 6 Medicina e Chirurgia d’Urgenza e Accettazione, AOU-Careggi, Florence, Italy; 7 Dipartimento di Diagnostica per Immagini, Azienda Usl Toscana Centro, Prato, Italy; 8 Neuroradiologia, Azienda USL Toscana Centro, Florence, Italy; 9 Otorinolaringoiatria, Arcispedale Santa Maria Nuova, IRCCS, Reggio Emilia, Italy; 10 Otorinolaringoiatria, Libero Professionista, Milan, Italy; 11 Pronto Soccorso e Medicina d’Urgenza, AUSL della Romagna, Ospedale Morgagni-Pierantoni, Forlì, Italy; 12 Medicina d’Urgenza, Ospedale Maria Vittoria, Turin, Italy; 13 Università degli Studi di Pisa, Pisa, Italy

**Keywords:** Vertigo, Dizziness, Nystagmus, Benign paroxysmal positional vertigo, Acute peripheral vestibular dysfunction, Ischemic stroke, Emergency Medicine, STANDING, HINTS

## Abstract

Acute vertigo is defined as the perception of movement of oneself or the surroundings in the absence of actual motion and it is a frequent cause for emergency department admissions. The utilization of medical resources and the duration of hospital stay for this kind of symptom is high. Furthermore, the efficiency of brain imaging in the acute phase is low, considering the limited sensitivity of both CT and MRI for diagnosing diseases that are the causes of central type of vertigo. Relying on imaging tests can provide false reassurance in the event of negative results or prolong the in-hospital work-up improperly. On the other hand, clinical examinations, notably the assessment of nystagmus’ features, have proven to be highly accurate and efficient when performed by experts. Literature data point out that emergency physicians often do not employ these skills or use them incorrectly. Several clinical algorithms have been introduced in recent years with the aim of enhancing the diagnostic accuracy of emergency physicians when evaluating this specific pathology. Both the ‘HINTS and ‘STANDING’ algorithms have undergone external validation in emergency physician hands, showing good diagnostic accuracy. The objective of this consensus document is to provide scientific evidence supporting the clinical decisions made by physicians assessing adult patients with acute vertigo in the emergency department, particularly in cases without clear associated neurological signs. The document aims to offer a straightforward and multidisciplinary approach. At the same time, it tries to delineate benchmarks for the formulation of local diagnostic and therapeutic pathways, as well as provide a base for the development of training and research initiatives.

**Supplementary Information:**

The online version contains supplementary material available at 10.1007/s11739-024-03664-x.

## Introduction

Acute vertigo, defined as the perception of movement of oneself or the surroundings in the absence of actual motion [1], is a frequent cause of emergency department [ED] admissions, constituting 2.1–3.6% of visits in the USA [2]. The utilization of medical resources and the duration of hospital stay for these patients surpass those associated with other similarly prevalent presentations [2, 3]. In Italy, the prevalence of ED admissions for vertigo is comparable, with a notable inclination towards requesting brain imaging tests; indeed, up to 70% of patients undergo such examinations in Italian EDs [4, 5]. However, the efficacy of brain imaging in the acute phase is low, considering the limited sensitivity of both CT and MRI for diagnosing strokes within the initial 12 h following the event [6]. CT exhibits a sensitivity of approximately 10% in detecting posterior circulation ischemic strokes [7], and the likelihood of a negative MRI is five times higher than that for a stroke occurring in the anterior circulation [8]. Relying on imaging tests can provide false reassurance in the event of negative results [9, 10], potentially prolonging the ED stay without commensurate enhancement in diagnostic accuracy [11, 12].

While diagnostic imaging tests have shown limitations in the initial assessment of patients with acute vertigo, clinical examinations, notably the accurate assessment of nystagmus, have proven to be highly accurate and efficient when performed by experts [13, 14]. Regrettably, literature data highlight that emergency physicians often do not employ these skills or use them incorrectly [15].

In an effort to bridge this knowledge and training gap, several clinical algorithms have been introduced in recent years, designed to enhance the diagnostic accuracy of emergency physicians when evaluating this specific pathology [5, 16–19]. Notably, both the 'HINTS' and 'STANDING' algorithms have undergone external validation, affirming their good diagnostic accuracy [15, 20]. Moreover, there have been recent publications of international guidelines dedicated to the management of patients with vertigo in the ED [21].

The objective of this consensus document is to furnish scientific evidence supporting the routine clinical decisions made by physicians assessing adult patients with acute vertigo, particularly in cases without clear associated neurological signs [referred to as isolated vertigo]. The document aims to offer a straightforward and multidisciplinary approach. Simultaneously, it seeks to establish benchmarks for the formulation of local diagnostic and therapeutic pathways, as well as provide a foundation for the development and implementation of training and research initiatives.

## Methods

The approach selected for defining the management of a patient with acute vertigo was not that of a systematic review but rather a consensus document. This decision stemmed from the scarcity of high-quality scientific production, specifically randomized and controlled studies, and the challenge of translating the subject matter into stringent scientific evidence. Consequently, the formulation of a consensus document was considered appropriate to articulate the collective opinion of the authors, selected as authoritative experts on the subject [refer to Supplementary Data “Methods”].

### Epidemiology in the emergency department

#### Frequent causes of *vertigo*

Isolated vertigo typically has a benign etiology and is often referred to an inner ear disorder. Nevertheless, it may occasionally indicate an underlying brain pathology, including a stroke [ischemic or hemorrhagic], a neoplasm, or a demyelinating disease [22, 23].

The diagnostic process is frequently challenging, given that patients may use the term "vertigo" to describe symptoms associated with various pathological conditions, such as orthostatic hypotension, postural instability, bradycardia, anxiety and panic disorders linked to hyperventilation, hypoglycemia, anemia, and electrolyte abnormalities [24]. Therefore, in addition to a thorough medical history, a precise clinical evaluation involving the study of nystagmus is often necessary.

Among the peripheral forms, the most prevalent cause is benign paroxysmal positional vertigo [BPPV] [25], characterized by short-term vertigo induced by head movements. BPPV can be identified through bedside diagnostic maneuvers like Dix-Hallpike and Pagnini-McClure [26]. The second most common cause of vertigo in the ED population is acute peripheral vestibular dysfunction [APVD] [27]. This term encompasses various conditions that are challenging to differentiate nosologically, including labyrinthitis, vestibular neuritis, and vascular vestibulopathy [28].

The prevalence of central forms varies significantly among studies. Some investigations conducted in ED settings have identified central causes in 3–6% of cases, with the majority attributed to stroke [2, 29, 30]. In contrast, according to other authors [31], central forms constitute nearly a quarter of patients with vertigo, but they also include transient vascular disorders of the posterior circulation, vestibular migraine [VM] [affecting approximately 1% of the population] [32], multiple sclerosis, tumors of the posterior cranial fossa, and toxic causes, including drugs such as antiepileptics [33].

## Clinical approach for differential diagnosis

### Medical history

#### General concepts

Concerning acute isolated vertigo the triage questions should primarily aim to ascertain whether the reported symptom can be categorized as peripheral vertigo or not.

The questions should include the evaluation of:

- *triggering factors*: for example, if symptoms arose following head movements, such as getting up or going to bed, or if there was a sudden onset without identifiable triggers.
- *duration*: if they last for a few seconds, minutes, hours, or days, and ascertain whether they are still ongoing or improving;
- *prodromes or associated symptoms:* Explore the presence of neurological and/or cardiovascular symptoms [e.g., headache, dysarthria, diplopia, dysphagia, chest pain, dyspnea, palpitations].;
- *ability to maintain an upright position*
- *cardiovascular risk profile*, i.e. history of diabetes mellitus, arterial hypertension, vascular disease.

In recent years, a growing amount of scientific evidence advocates for an approach centered on "timing" and "triggers" rather than focusing solely on the quality of symptoms, such as distinguishing between objective vertigo and disequilibrium [21].

This approach allows for the identification and differentiation of three potential vestibular syndromes:

- Acute vertiginous syndrome [AVrS] [lasting many hours to days];
- Episodic vertiginous syndrome [EVrS] [lasting a few minutes to hours] is further categorized into:
- Spontaneous EVrS [s-EVrS]
- Triggered EVrS [t-EVrS]

It's noteworthy to mention that the English literature employs a nomenclature that we find potentially misleading. It literally refers to an "Acute Vestibular Syndrome" and an "Episodic Vestibular Syndrome." We believe that using the term "Vestibular" to describe a symptomatic picture, as outlined below, in addition to being non-topodiagnostic, i.e., not indicative of a specific lesion in the peripheral or central vestibular system, it is not appropriate. Hence, we have opted to replace the term "vestibular" with "vertiginous" in these definitions, as we find it to be a more appropriate descriptor for a symptom.

#### Acute vertiginous syndrome

AVrS is characterised by a sudden onset of vertigo lasting more than 24 h and it is accompanied by autonomic symptoms such as nausea and vomiting, along with postural instability, typically in the absence of auditory symptoms. It may arise from either a sudden dysfunction of a peripheral vestibular receptor [APVD] or a dysfunction of the central vestibular structures within the central nervous system [CNS].

The most frequent peripheral cause of AVrS is represented by APVD, generally attributed to viral neuritis [VN] [34–36] [for further information see Supplementary Data “Medical History”].

Regardless of the cause for APVD, the clinical features of this condition result from an isolated lesion of the peripheral vestibular receptor [labyrinth and/or vestibular nerve]. This leads to the absence of auditory symptoms or CNS signs/symptoms.

However, as mentioned earlier, AVrS can also be the initial sign of an ischemic stroke, particularly involving the brainstem or cerebellum, Menière's disease [MD] or vestibular migraine [VM].

In AVrS of peripheral origin, symptoms are not associated with other neurological deficits. The presence of sudden hearing loss raises the possibility of an ischemic syndrome of the anterior inferior cerebellar artery [AICA]. The AICA gives rise to the internal auditory artery with its cochlear branch, making this syndrome a consideration, especially in patients with known cardiovascular risk factors.

## Classification

### Episodic vertiginous syndrome

EVrS encompasses transient and recurrent episodes of vertigo, varying in duration from seconds to minutes or hours. In certain instances, the episodes of vertigo can be numerous and may be spaced well apart over time. Two distinct types are acknowledged: those without triggering factors, termed spontaneous or non-positional, and those with triggering factors, referred to as provoked or positional.

### Spontaneous episodic vertiginous syndrome [s-EVrS]

The duration of spontaneous episodic vertigo typically spans from minutes to a few hours. Upon admission to the emergency department [ED], patients with these symptoms are often asymptomatic, and the diagnosis relies predominantly on the medical history. The frequency of these vertigo attacks can vary, occurring from several times a day to once a month, contingent on the underlying pathology.

s-EVrS is most associated with benign conditions such as VM, MD, reflex syncope, and panic attacks. Less common but potentially dangerous causes include cerebrovascular diseases [vertebrobasilar TIA] and non-vestibular conditions [pseudo-vertigo], such as cardio-respiratory diseases [cardiac arrhythmia, pulmonary embolism, and unstable angina], endocrinopathies [hypoglycemia], and intoxications [carbon monoxide]. [for further information see Supplementary Data “Medical History”].

### Triggered episodic vertiginous syndrome [t-EVrS]

Episodes of vertigo can be triggered by specific actions or events, with the most common triggers being head movements and changes in body position. For instance, standing up from a sitting or lying position, tilting the head in the shower to wash hair, or turning over in bed are frequent triggers. Less common triggers include intense sounds or the Valsalva maneuver. The duration of each episode of dizziness varies, lasting from a few seconds to minutes, contingent on the underlying cause. The most frequent causes of t-EVrS include BPPV and orthostatic hypotension. As some forms of vertigo can be repetitive and nausea may persist between individual episodes, it is possible for some patients to overestimate the overall duration of the episode. [for further information see Supplementary Data “Medical History”].

Among other dangerous causes, it is crucial to consider forms of CPPV, which may arise from expansive lesions in the posterior cranial fossa. This condition is typically associated with other neurological signs and symptoms. Additionally, non-benign causes of orthostatic hypotension, such as internal hemorrhage [refer to the chapter "Focused Physical Examination"], should be considered.

### Focused physical examination

#### General concepts

The physical examination in the Emergency Department [ED] is typically conducted concurrently with history taking, often employing a 'head-to-toe' approach. The objective is to identify signs that may either support or challenge the diagnostic hypothesis formulated during the history-taking process. [refer to the chapter "Medical History"].

**Fig. 1:**
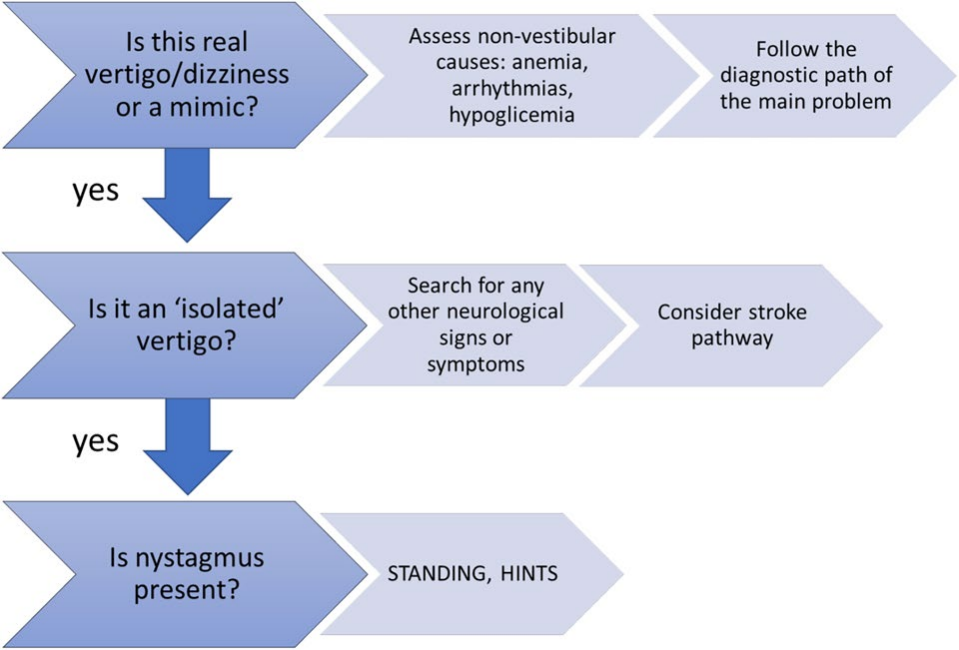
Essential steps of clinical evaluation

During this first clinical evaluation, we proceed through three ordered phases (Fig. 1):

- Exclude the presence of warning signs or symptoms ('red flags') of serious non-vestibular diseases that can mimic vertigo (see Table 1)
- Confirm what was hypothesized at triage, i.e. that it is isolated vertigo, without other associated neurological signs
- Especially in case of negativity of the two previous phases, the nystagmus examination is useful, preferably through validated algorithms.

**Table 1:** Differential diagnosis: possible causes of pseudovertigo/mimics

Differential diagnosis: pseudovertigo			
Disease	Focused anamnesis	Clinical signs	Diagnostics
Cardiogenic or arrhythmic pre-syncope	Transient hemodynamic cerebral hypoperfusion	Bradycardia and/or symptomatic cardiac pause Sustained tachyarrhythmia	12-lead EKG EKG monitoring
Hypoglycemia	Known diabetes mellitus + fasting + improper antidiabetic intake	Mental confusion, sweating with diaphoresis	Spot glycemic stick
Severe anemia	Acute blood loss, martial deficiency	Pallor Tachycardia Melena	Blood count [venous sampling]
Pre-syncope due to orthostatic hypotension	Transient dizzy episode sustained by cerebral hypoperfusion	Orthostatic hypotension when assuming an upright posture [measurements at 0–1-3–5 min]	Orthostatic hypotension tests
Electrolytes disorders [Na, K, Ca, Mg]	Chronic diuretic therapy Chronic therapy with chelating or proton pump inhibitors	Asthenia Hypotension Central nystagmus [severe forms of hypo-Mg]	Serum electrolytes [venous sampling]
Alcohol intoxication	Known alcoholism	Ethyl breath, ataxic gait, dysphoria, spider nevi	Ethanolemia
Hyperventilation	Anxiety-depressive disorder, panic attack, psychotic disorders Pregnancy	Large shaking oscillations, somatizations and/or psychotic manifestations	Normoxic hypocapnia [arterial blood gas analysis]
Epilepsy	History of partial or generalized epilepsy, risk factors, epileptogenic factors	Tics, lateral bite, sphincter release, post-ictal state	Lactate EEG
CO poisoning	Consensual disorder between cohabitants, housing problems, long stay in closed places, fires	Headache Influenza-like illness, tachycardia	CO-Hb [arterial blood gas analysis]
Intracranial hypotension	Associated with postural headache, recent lumbar puncture	Headache, nausea and vomiting, tinnitus/muffling, ± cranial nerve deficit	Neuroimaging

#### Neurological examination

In patients presenting with an acute onset of vertigo syndrome, a comprehensive neurological examination is essential. Utilizing the following checklist can help identify: (1) language disorders (dysarthria, aphasia); (2) ocular motility disorders; (3) disorders of the bulbar cranial nerves (glossopharyngeal nerve and vagus nerve); (4) Bernard-Horner syndrome; (5) visual field defects; (6) motor deficits [Mingazzini maneuvers, resistance tests]; (7) sensory deficits (tactile, pain, thermal, and deep); (8) cerebellar deficits; (9) plantar cutaneous reflex in extension (Babinski sign). The presence of at least one of these signs should be considered indicative of a potentially central origin of vertigo [for further information see Supplementary Data “Neurological examination”].

One of the main steps is the evaluation of balance and gait. We reported a simple method to use in everyday clinical practice (Table 2) according to Carmona et al. [37]. Grade 0 and 1 showed ‘per se’ a high negative predictive value to exclude stroke (> 90%). Concerning vascular etiology, which predominates among central forms, neurological signs, and symptoms vary based on the specific vascular district involved (Table 3). The clinical evaluation of ocular motility and of nystagmus are other crucial nodes of neurological evaluation in patients with dizziness/vertigo, often misinterpreted by non-dedicated specialists. For this reason, we try to give the reader a more detailed and plane description of the relevant elements in everyday clinical practice.

**Table 2:** Assessment of gait unsteadiness [adapted from Carmona S et al. 2016][37]

Severity of gait unsteadiness	Definition	Positive predictive value of ataxia grade for stroke
Grade 0	Normal	0% [*n* = 0/5] had stroke
Grade 1	mild to moderate imbalance with walking independently	7% [*n* = 3/42] had stroke
Grade 2	severe imbalance when standing or cannot walk without support	28% [*n* = 11/39] had stroke
Grade 3	falling at upright posture/inability to stand unaided	100% [*n* = 28/28] had stroke

**Table 3:** Cerebellar vascular syndromes [adapted from Ropper and Samuels, 2019]

AICA stroke	PICA stroke	SCA stroke
[a] Vertigo	[a] Vertigo	[a] Ataxia [+ + of the trunk]
[b] Ipsilateral dysmetria	[b] Ipsilateral dysmetria	[b] Dysarthria
[c] Bernard-Horner syndrome	[c] Bernard-Horner syndrome	[c] Contralateral pain and temperature hypoesthesia
[d] Ipsilateral facial hypoesthesia, contralateral pain and thermal hypoesthesia	[d] Ipsilateral facial hypoesthesia, contralateral pain and thermal hypoesthesia	[d] Nausea and vomit
[e] Hearing loss and tinnitus	[e] Paralysis of the palatine velum Dysphagia	
[f] VI° and VII° cranial nerve palsy	[f] Nausea and vomit	

*AICA* Anterior Inferior Cerebellar Artery, *PICA* Posterior Inferior Cerebellar Artery, *SCA* Superior Cerebellar Artery

#### Clinical examination of ocular motility

This includes the analysis of slow pursuit movements [also known as smooth pursuit], saccades, and optokinetic nystagmus. [refer to Supplementary Data “The clinical examination of ocular motility”].

## The evaluation of nystagmus

The observation and analysis of nystagmus are fundamental in the evaluation of patients with acute vertigo/dizziness.

### Nystagmus observation techniques

**Fig. 2:**
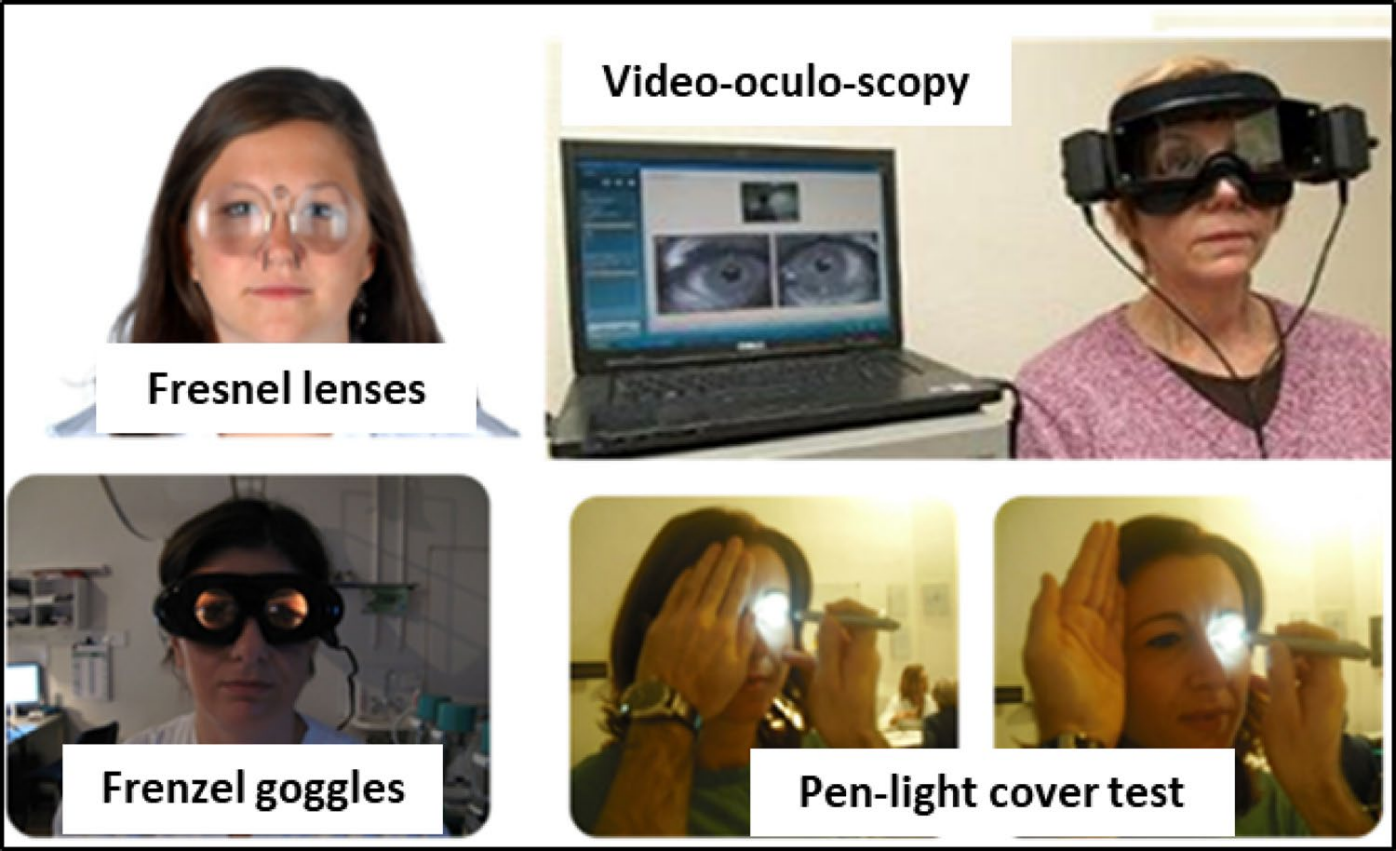
Techniques for detecting nystagmus without fixation

Observing nystagmus necessitates both fixation and non-fixation conditions, as fixation tends to mask certain nystagmus types, particularly those originating peripherally. Various techniques (Fig. 2) can be employed to eliminate fixation, with the Frenzel goggles being the simplest and most practical option [38–40].

#### Nystagmus search

When assessing for nystagmus, it is essential to conduct examinations in the primary position of gaze, both with or without fixation. This should be done with the patient looking straight ahead while keeping the head still, in both supine and sitting positions. The examiner should carefully observe the following aspects:

- (1) Determine whether nystagmus is present;
- (2) Identify the plane on which the nystagmus beats, whether it is horizontal or vertical;
- (3) Determine the direction of the nystagmus, such as right, left, upwards, downwards, rotary, or oblique;
- (4) Observe the temporal trend,
- (5) Evaluate how the nystagmus responds to changes in gaze position. Ask the patient to move their gaze [without moving their head] to the right, left, up, and down [in this last position, the examiner will just lift the eyebrows, and maybe even a little eyelids];
- (6) Slowly change the patient's head position and observe how the nystagmus responds:
- the *supine position*, the one in which the patient with acute vertigo usually arrives in the ED lying on a stretcher and in which the nystagmus is evaluated in the primary position [see above];
- the *right side position* [or right lateral-rotated head position]: The patient remains in a supine position, and the head is rotated 90° to the right. Alternatively, if the patient has difficulty rotating the head, the entire body is rotated 90° to the right while keeping the head on axis;
- the *position on the left side* mirrors the previous one;
- *the sitting position.*

Two other positions that are reached quickly are defined as Dix-Hallpike “positionings”, named as the two authors, Margaret Dix and Charles Hallpike, who described them for the first time. [41] (see the following chapter Benign Paroxysmal Positional Vertigo, page 14).

### Clinical tests

We outline some useful tests for the investigation of patients with vertigo in the ED.

#### Head impulse test

**Fig. 3:**
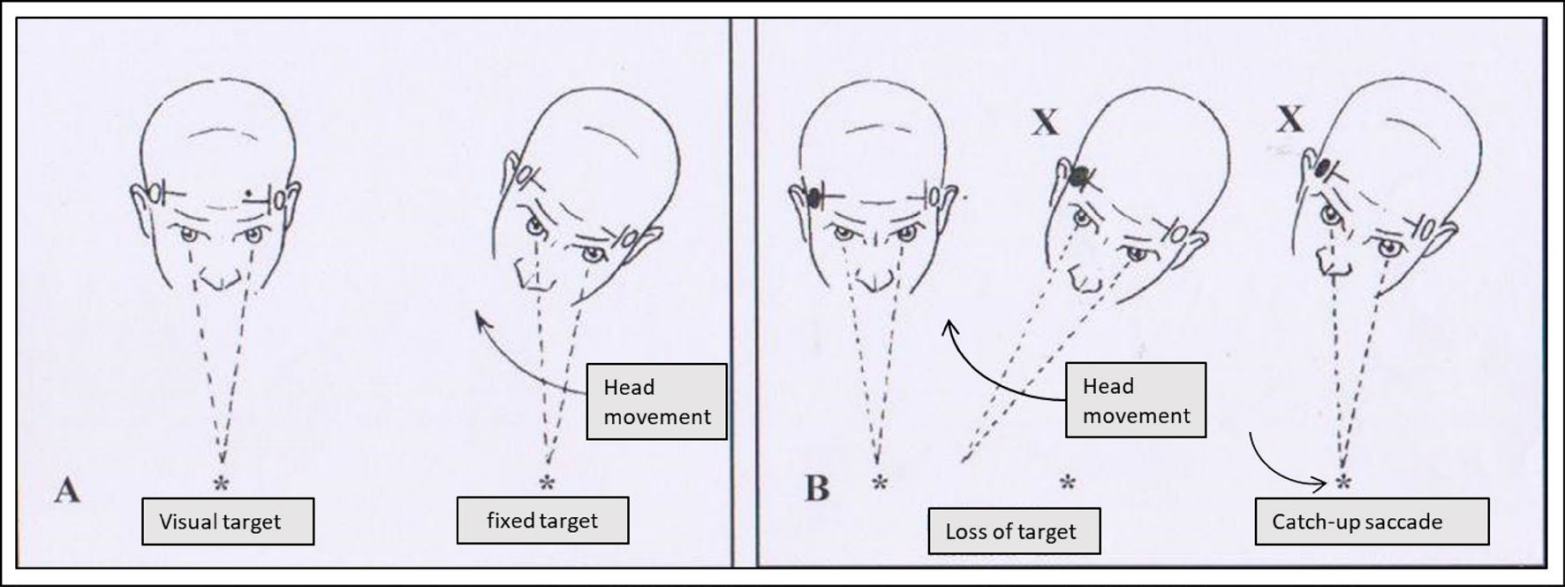
HIT [Head Impulse Test] **A** negative HIT, normal function of the right lateral canal: suspected central disease. **B** positive HIT, probable peripheral pathology due to right APVD

The Head Impulse Test [HIT] stands as a pivotal and often decisive clinical examination. In a normal scenario, when a subject focuses on a target [e.g., the operator's nose] and their head is swiftly rotated in one direction, the eyes exhibit a compensatory movement in the opposite direction [counter-rotation] to maintain fixation on the target (Fig. 3A). This counter-rotation is facilitated by the Vestibulo-Oculomotor Reflex [VOR], relying on the intact functionality of the semicircular canals within the inner ear. Should damage occur to the lateral semicircular canal on the side toward which the head is rotated, the eyes will exhibit a diminished or absent counter-rotation when the head moves toward the affected ear. Consequently, the patient loses sight of the target momentarily, followed by a rapid "saccadic" movement to swiftly regain focus on the fixed object [the nose of the examiner] (Fig. 3B). This corrective movement is directed toward the healthy ear.

The positivity of HIT toward one side, as opposed to the other, serves as a highly indicative sign of labyrinthine damage and, consequently, peripheral origin of vertigo. The side of positivity corresponds to the direction in which the patient's head is rotated, not the direction of the subsequent recovery saccadic movement.

#### Cover test

In certain cases of vertigo, visual inspection of a patient may reveal a vertical misalignment of the eyes, with one eye positioned higher and the other lower. A diagnostic test involves covering one eye at a time and observing the movement of the uncovered eye. If, upon covering the lower eye, the higher eye moves downward, and vice versa, the test is considered positive. This finding is highly indicative [with a positive predictive value exceeding 90%] of central pathology. However, if a noticeable realignment of the eyes is not observed, the test is deemed negative and does not provide conclusive evidence regarding the location of the underlying damage [16].

The Head shaking test and other useful bedside test are reported online. [refer to Supplementary Data” Additional Diagnostic Maneuvers”].

## Frequent diseases in the emergency department

The identification of prevalent peripheral clinical diseases facilitates the identification of patients eligible for referral to specialized audio-vestibular outpatient evaluation. This, in turn, substantially diminishes the need for additional diagnostic investigations in the ED.

### Benign paroxysmal positional vertigo

#### Epidemiology

BPPV stands out as the most prevalent labyrinthine disease, characterized by short and paroxysmal episodes. While there exist various forms of BPPV, this document specifically focuses on the "classic" variant involving the posterior semicircular canal [PSC] and the lateral semicircular canal [LSC] in its geotropic manifestation [42–44]. The apogeotropic form of LSC will be briefly mentioned in the online material, while the apogeotropic forms of PSC and those affecting the anterior semicircular canal (ASC) will be excluded due to less defined and more variable nystagmus patterns, sometimes resembling patterns of potential ‘central’ origin.

BPPV often undergoes spontaneous resolution prior to medical observation. It exhibits an annual recurrence rate of 20%, with an estimated prevalence of approximately 50% among patients accessing the ED for isolated vertigo, as indicated by a recent Italian study [5].

In 70%-80% of cases, BPPV is attributed to the PSC, predominantly affecting the right side. The second most prevalent form involves the LSC, with an estimated incidence ranging from 5 to 30%. Less common variations encompass the ASC, constituting approximately 2%, and multi-canal forms, typically concurrent or subsequent, predominantly of post-traumatic origin.

Incidence rates escalate with age, and while the etiology is commonly idiopathic, recent studies have identified associations with female gender, reduced serum levels of vitamin D, migraine, head trauma, and an elevation in cholesterolemia.

### BPPV-PSC

#### Symptoms

Symptoms of BPPV-PSC manifest as brief, varying intensity episodes of dizziness provoked by movements in the vertical plane, such as arising or lying down in bed. These episodes typically have a short latency period, ranging from 0 to 15 s. Vertigo follows a paroxysmal pattern, escalating rapidly, reaching a plateau, and subsequently diminishing. It may be accompanied by nausea and/or vomiting. While a degree of instability may persist when standing for prolonged periods, BPPV is categorized as one of the triggered episodic vertiginous syndromes [t-EVrS].

#### Nystagmus

**Fig. 4:**
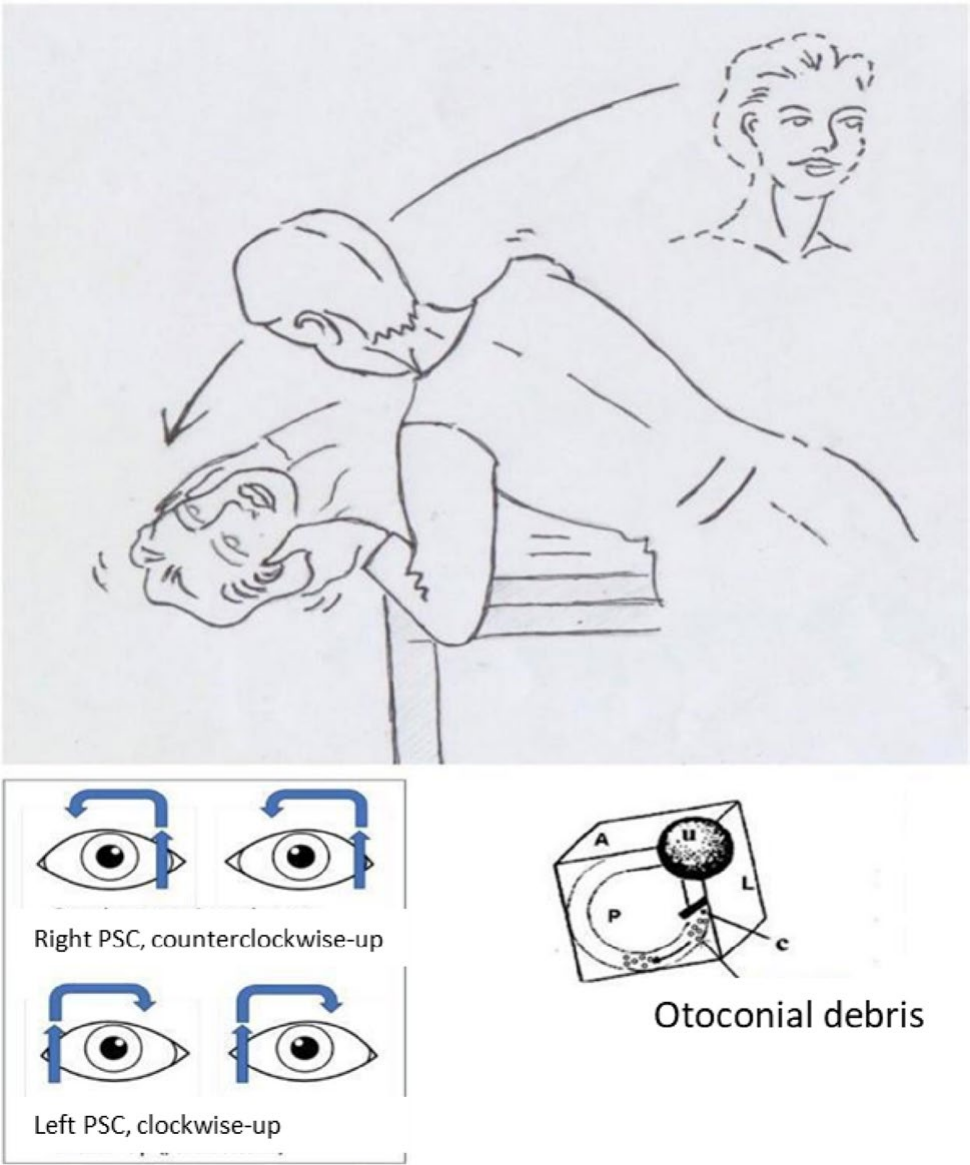
*The right Dix-Hallpike maneuver*. The patient begins in a seated position with legs on the table. The examiner gradually rotates the patient's head approximately 45° to the right and quickly transitions him to a supine position, with the head hyperextended out of the table. In cases of significant dorsal spine kyphosis, commonly observed in older patients, simple supine positioning achieves the desired hyperextension of the head. T*he left Dix-Hallpike maneuver* mirrors the aforementioned procedure. [PSC: Posterior Semicircular Canal, A: plane of the anterior canal, P: plane of the posterior canal L: plane of the lateral canal, U: utricle, C: dome]

The hallmark clinical sign that characterizes BPPV is the nystagmus that accompanies the symptoms. Nystagmus is absent when the patient is at rest, necessitating the clinician to actively induce manoeuvres to elicit it, particularly in the ED setting. The Dix-Hallpike manoeuvre (Fig. 4, upper panel) represents the most accurate diagnostic approach. The rapid phase of the nystagmus is directed towards the right shoulder (counterclockwise-up for the observer) for right-sided stimulation and towards the left shoulder (clockwise-up for the viewer) for left-sided stimulation (Fig. 4, lower-left panel). A latency period is often observed, likely attributed to material overcoming resistance within the ampoule and/or canal before movement occurs. The nystagmus is paroxysmal, intensifying rapidly, reaching a peak within seconds, and then diminishing.[for further details refer to Supplementary Data ”Benign Paroxysmal Positional Vertigo”].

### BPPV-LSC

#### Symptoms

BPPV-LSC manifests as vertigo triggered by horizontal plane movements while in the supine position [e.g., turning from supine to the side or from side to side, such as turning over in bed]. Vertigo in BPPV-LSC tends to be more intense and prolonged compared to BPPV-PSC. It recurs with each movement and is frequently accompanied by nausea and vomiting. Unsteadiness when standing is less common than in BPPV-PSC. Occasionally, the resolution is immediate, with symptoms disappearing upon rotating in bed.

#### Nystagmus

**Fig. 5:**
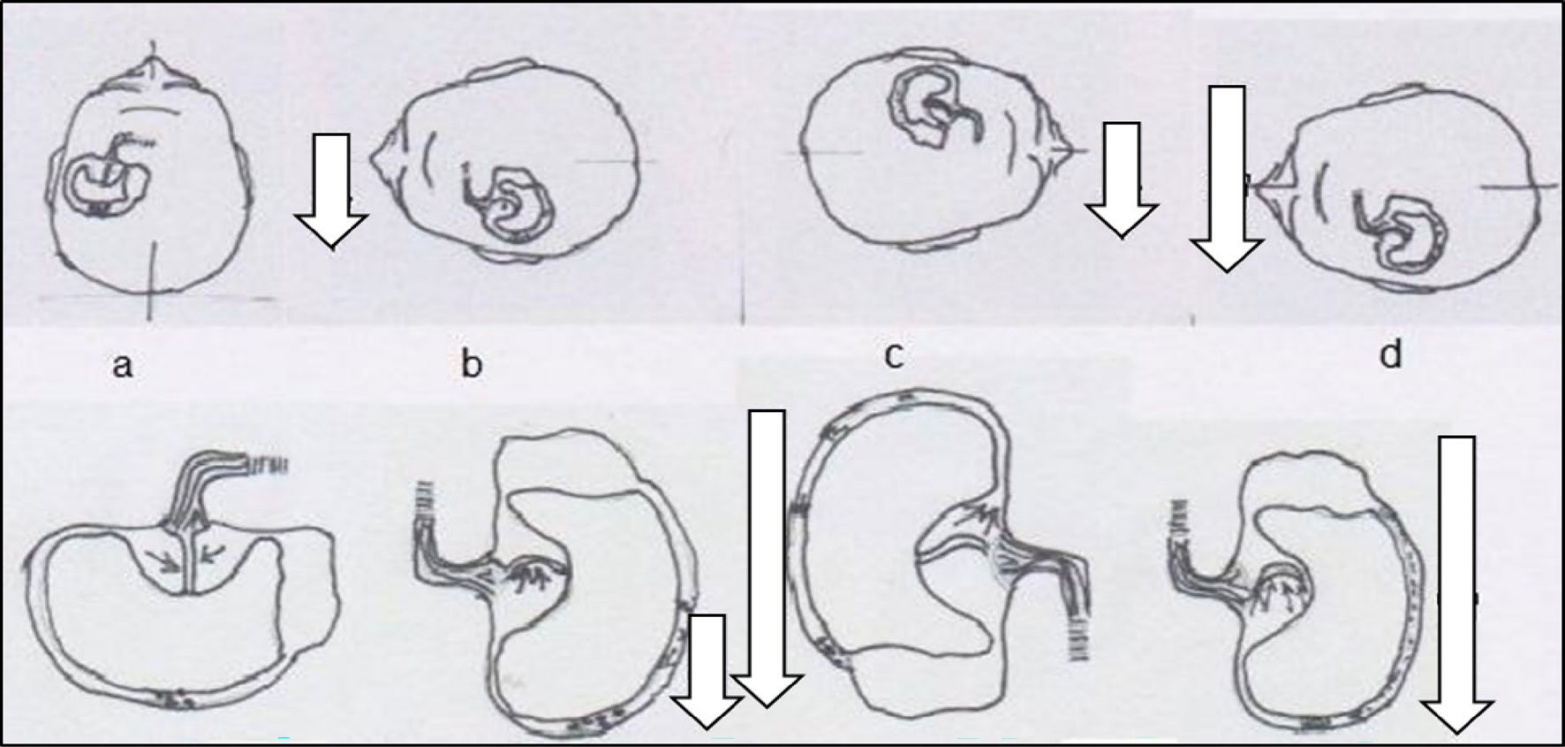
BPPV-LSC in geotropic form. The figure presents head positions at the top and the corresponding excursion of otoconial debris at the bottom. Arrows above denote the direction and intensity of nystagmus, while below indicate the amplitude of debris movement. **a** The patient in a supine position with a stable head exhibits an absence of nystagmus. **b** Upon a 90° rotation of the head to the left, there is an ampullipetal movement of otoconial debris, resulting in a moderately intense left geotropic nystagmus. **c** A 180° rotation of the head to the right induces a larger, ampullifugal movement of inhibitory otoconial debris, accompanied by a right geotropic nystagmus of medium intensity. **d** A 180° movement of the head to the left leads to a broader excursion of otoconial debris in the ampullipetal direction, causing a vigorous left geotropic nystagmus

While in a sitting position or supine for at least 5 min, nystagmus is typically absent. When the patient turns their head toward the affected ear or lies on the same side, it results in ‘geotropic’ nystagmus, characterized by the rapid phase beating towards the affected ear and, consequently, towards the ground (see Fig. 5b). The subsequent transition to the opposite side induces a ‘geotropic’ nystagmus with a rapid phase consistently directed towards the ground (Fig. 5c). The observing clinician will note a right nystagmus when the patient lies on the right side and a left nystagmus when lying on the left side (Fig. 5d). A more pronounced nystagmus is typically observed when the patient lies on the affected side. It is crucial that head positioning on both sides is executed with similar amplitudes and speeds. [for further details, apogeotropic form, refer to Supplementary Data ”Benign Paroxysmal Positional Vertigo”].

## Acute peripheral vestibular dysfunction

### Epidemiology

The sudden loss of function in one of the two peripheral vestibular systems, commonly referred to as Acute Peripheral Vestibular Dysfunction [APVD], is often denoted as VN. However, it's important to note that the term "vestibular neuritis" implies an unproven pathological alteration, specifically inflammation of the vestibular nerve. Despite this, VN is widely recognized as one of the most prevalent disorders affecting the peripheral vestibular system. It is regarded as the third most common peripheral vestibular disease in terms of presentation frequency in the general population [after BPPV and MD] [45]. While the average age of onset is commonly placed between 30 and 60 years [46], there appears to be no gender or affected side prevalence. The recurrence rate seems relatively low (10%) [47]. Bilateral APVD appears to be exceptionally rare [48, 49]. The estimated prevalence of APVD in patients presenting to the ED with isolated vertigo is 15.9%, according to recent Italian research [5], placing it in second place among causes of vertigo/dizziness in the ED.

### Symptoms

The symptomatology aligns with AVrS, characterized by a sudden-onset vertigo lasting more than 24 h. It is accompanied by neurovegetative symptoms such as nausea and vomiting, along with postural instability, generally in the absence of auditory symptoms.

### Objective findings and nystagmus

Diagnosis is characterized by the so-called "harmonic syndrome," including:

- Spontaneous horizontal-torsional nystagmus beating away from the affected side, maintaining a consistent direction regardless of gaze orientation [50].
- Horizontal head impulse test [HIT] with refixation saccades following a head impulse directed toward the affected side, resulting in a positive HIT toward the affected side [51].
- Tonic-segmental deviations of the head and trunk toward the affected side [lateropulsion] during evaluation in an upright position [static phase] and/or during ambulation [dynamic phase] [52].

The evaluation of nystagmus, better without visual fixation, serves as a pivotal element. In cases of APVD, spontaneous nystagmus will be always present, characterized by a biphasic ocular movement evident when the head is stationary [regardless of the evaluation position, supine or seated]. Peripheral APVD nystagmus, linked to damage to the semicircular canals, particularly the lateral canal, typically manifests as horizontal rotatory. The fast phase is directed towards the healthy side [contra-lesional]. This nystagmus should consistently be unidirectional, maintaining the same direction and plane irrespective of head and gaze positions. [refer to Supplementary Data” Acute Peripheral Vestibular Dysfunction”]. The nystagmus is *persistent*, enduring as long as it is observed, and maintains a *stable* character over time, with its speed remaining essentially unchanged. In the acute phase, fixation inhibition may be only partial (observable even under fixation). Before attributing nystagmus with reasonable certainty to a labyrinthine origin, indicative of unilateral APVD, the Head Impulse Test (HIT) should be conducted (see chapter “Clinical evaluation of ocular motility”, page 12). This test verifies the absent or reduced function of VOR on the affected side. A positive result after a rapid (impulsive) rotation towards the affected side, opposite to the direction of spontaneous nystagmus, suggests that the tested labyrinth is incapable of generating an effective VOR. Consequently, it is highly probable that the patient has a problem of a peripheral vestibular nature. [for details on Ocular Tilt reaction, refer to Supplementary Data”Acute Peripheral Vestibular Dysfunction”].

During the acute phase of APVD and in the initial days of symptom onset, there is almost invariably a static [evaluated with the Romberg test] and dynamic [explored using the Unterberger test] instability due to the loss of the vestibulo-spinal reflex [VSR] on the affected side. This condition is easily objectified, as the patient, while able to maintain an upright stance with open eyes, tends to deviate towards the affected side, which intensifies upon closing their eyes [Romberg test].

Moreover, when asked to perform a marching-in-place with arms outstretched and eyes closed [Unterberger test], the patient tends to deviate towards the affected side. The patient can usually maintain an upright stance and/or walk unassisted.

## Meniere's syndrome

Given its low prevalence in ED, we have chosen not to delve into the details of this pathology and instead direct the reader to specialized texts [53, 54].

### Central nervous system disorders

#### Epidemiology

In a survey conducted by the Emergency and Urgent Neurology Association in 93 Italian hospitals, it emerged that out of 4609 patients who presented themselves to the emergency department in a single day, 683 neurological consultations were requested [15%]. Approximately 8% of these consultations were sought for vertigo.

Among the possible causes of acute vertigo, disorders of the posterior circulation constitute one of the most common central forms. A study by Royl et al. [55] highlighted that among patients presenting to the emergency department with vertigo, the final diagnosis was acute cerebrovascular pathology in 6% of cases. Lee et al. [24] demonstrated that out of 240 consecutive patients with a final diagnosis of cerebellar stroke, 10.4% presented to the ED with acute vertigo without other associated neurological signs.

Isolated vertigo can also be associated with a form of migraine known as vestibular migraine [VM]. The prevalence of this disease is unknown, as the described symptoms may overlap with those of other pathologies. However, it is estimated that 1–3% of the general population suffers from it [56]. The diagnosis of VM is based on criteria defined by the International Headache Society and the Bárány Society (Table 4).

**Table 4:** Diagnostic criteria for vestibular migraine according to the international headache society [IHS] 2018

A. At least five episodes that meet criteria C and D
B. Current or past history of 1.1 Migraine without aura or 1.2 Migraine with aura
C. Vestibular symptoms of moderate or severe intensity lasting between 5 min and 72 h
D. At least half of the episodes are associated with at least one of the following three migraine characteristics:
1. Headache with at least two of the following four features: (a) Unilateral pain (b) Throbbing pain (c) Moderate or severe pain (d) Pain aggravated by routine physical activity 2. Photophobia and phonophobia 3. Visual aura
E. Not better classified by another ICHD-3 diagnosis or by another vestibular disorder

#### Symptomatology

Different pathologies of CNS origin can present with vertigo, such as inflammatory, demyelinating, neoplastic diseases, substance use, metabolic alterations, and neurodegenerative conditions, although they will not be individually detailed in this context. Overall, we can assume that central forms may manifest as vertigo/disequilibrium, either in the form of an AVrS or episodic forms, whether provoked or spontaneous. In a patient with acute vertigo, the presence of associated neurological signs or symptoms [see *medical history* and *physical examination*] should guide toward a centrally originated pathology. In the case of isolated vertigo, careful observation of gait and nystagmus can still provide useful elements for differential diagnosis. While we do not aim to present a comprehensive treatise on oto-neurology, we believe that describing some more typical patterns of nystagmus of central origin can be helpful [please refer to Supplementary Data “Nystagmus patterns of central origin”].

## Diagnostic algorithms: HINTS, STANDING, and Titrate

Over the years, numerous clinical tests have been evaluated to distinguish acute peripheral vertigo from acute central vertigo. However, none of them, when considered individually, has proven capable of allowing an accurate differential diagnosis. For this reason, in the last decade, several diagnostic algorithms composed of multiple tests have been proposed. Below are the most reported tests and diagnostic algorithms in the literature.

The first bedside diagnostic algorithm is **HINTS** (**H**ead **I**mpulse Test, **N**ystagmus, **T**est of **S**kew) [16]: an otoneurological examination comprising three bedside tests, exclusively used in patients with AVrS. In the validation study, recruited patients had at least one risk factor for stroke [smoking, hypertension, diabetes mellitus, dyslipidemia, atrial fibrillation, hypercoagulability, recent head trauma, or previous stroke or myocardial infarction]. After applying the algorithm, all patients underwent brain MRI, and those with suspected peripheral pathology underwent caloric tests.

The authors define HINTS as "benign," suggesting a peripheral lesion, if the nystagmus is unidirectional, the Head Impulse Test [HIT] is positive, and there is no skew deviation. **HINTS is deemed "dangerous"** indicating a central form, if at least one of the following is present:

- Negative HIT
- Pure vertical or torsional spontaneous nystagmus or gaze-evoked nystagmus [variable-direction nystagmus]
- Positive cover test

Subsequently, the authors introduced a fourth step, the bedside assessment of hearing loss, hence the term HINTS "plus" [18]. Its presence may suggest a vascular origin due to the involvement of the anterior inferior cerebellar artery [AICA]. HINTS achieves sensitivity values of 99% [ability to identify central vestibulopathy] and specificity of 97% [ability to exclude stroke when absent], values superior to DWI-MRI performed in the first 24–48 h.

**Fig. 6:**
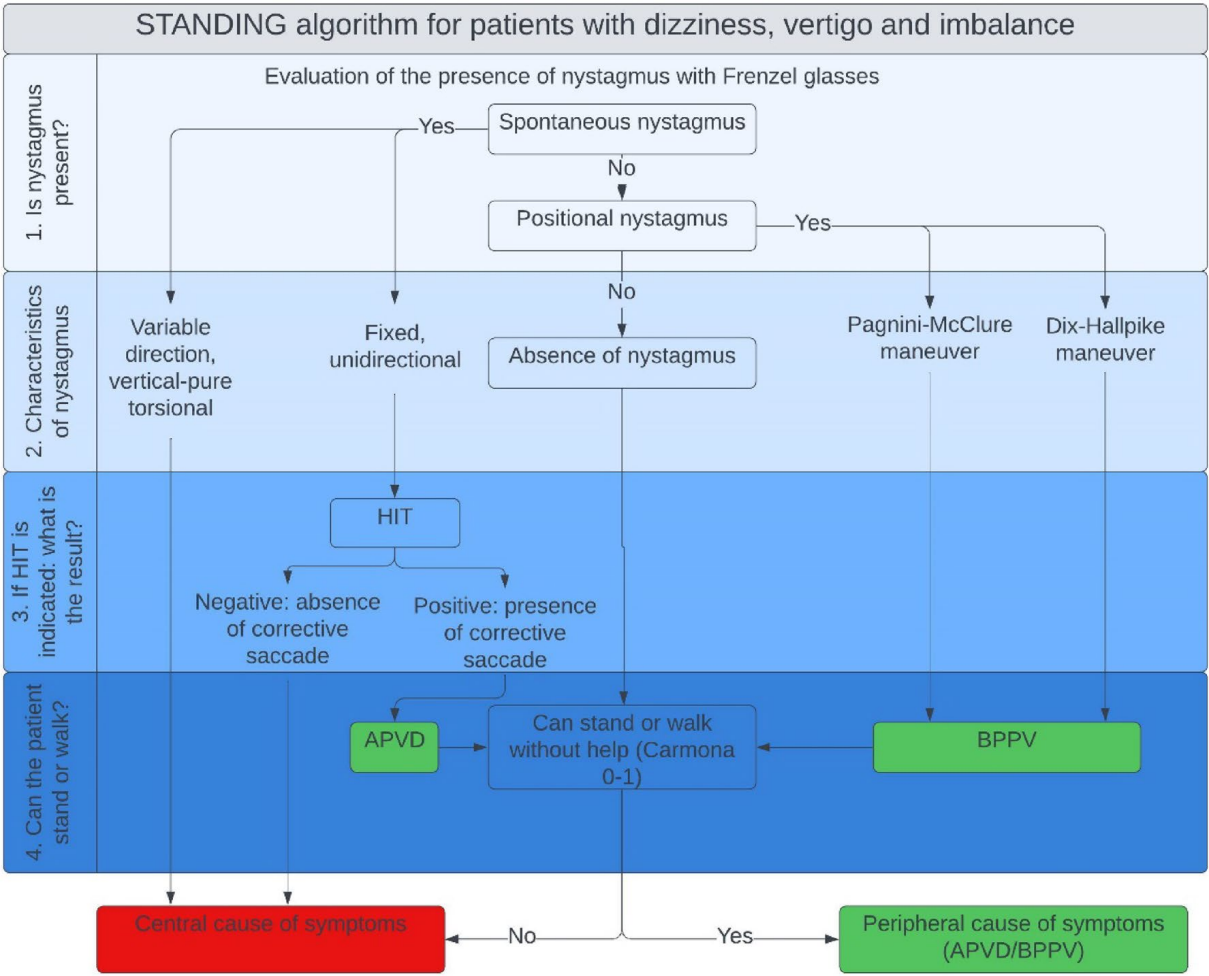
STANDING algorithm. HIT: Head Impulse Test; APVD: Acute Peripheral Vestibular Dysfunction: BPPV: Benign Paroxysmal Peripheral Vertigo

Another proposed and validated diagnostic algorithm is **STANDING** [**S**pon**TA**neous **N**ystagmus, **D**irection, Head **I**mpulse Test, Standi**NG**] (Fig. 6). It was developed through collaboration between vestibologists and emergency physicians and involves a learning period conducted by a physician experienced in evaluating patients with vertigo [4, 5]. It is a structured consecutive four-step algorithm based on the bedside signs described earlier, with the addition of some maneuvers performed at the patient's bedside [refer to Supplementary Data “Diagnostic algorithm”].

STANDING, conducted at the patient's bedside, has shown high reliability and accuracy in detecting the presence of central vestibulopathy in an unselected population of patients presenting to the emergency room with acute vertigo. The agreement between the assessment performed by emergency physicians using STANDING and that by a vestibular specialist [considered the reference standard] was quite high, with a Cohen's K of 0.86 [and a concordance rate of 95.6%]. The STANDING algorithm has recently been externally validated in two studies published by a group of emergency physicians in Paris [20, 57]. Both algorithms offer an accurate yet simplified assessment of nystagmus. While the HINTS algorithm should be applied to patients with AVrS and spontaneous nystagmus, the STANDING algorithm is suitable for evaluating all patients with isolated vertigo, including positional forms. Notably, the STANDING algorithm encompasses HINTS [except for the cover test], assessment of positional nystagmus, and evaluation of upright position and gait, enabling an initial differential diagnosis even for patients reporting vertigo with or without spontaneous or positional nystagmus. It is evident that both algorithms propose a diagnostic process primarily focused on nystagmus evaluation, which should be integrated by a targeted anamnestic and objective assessment of other organs and systems, particularly the neurological and cardiovascular systems.

The **TI.TR.A.T.E.** (**TI**ming, **TR**iggers, **A**nd **T**argeted **E**xamination) is a new diagnostic approach aimed at determining the probable etiology of acute vertigo, categorizing patients into four groups [17]. [refer to Supplementary Data “Diagnostic algorithms”].

## Diagnostic imaging

In recent years, there has been a consistent rise in requests for radiological examinations in the ED. Particularly, in the United States, from 1994 to 2015, neuroimaging requests escalated by 660% [58]. A similar trend is observed in Italy, where requests for neuroimaging in EDs continue to increase. Remarkably, up to 70% of patients with acute vertigo undergo urgent cranial CT or brain MRI [4, 5].

Imaging diagnostic modalities employed in patients with acute vertigo in the ED encompass CT, including CT Angiography [CTA] for cerebral and neck vessels, and CT Perfusion [CTP]. Additionally, MRI with Diffusion-Weighted Imaging [DWI] and Perfusion Imaging [PWI] techniques are utilized.

Key queries arising in the management of patients with acute vertigo include:

- Determining which patients necessitate neuroimaging
- Assessing whether a CT scan alone suffices or if an MRI is also required
- Deciding on the urgency of neuroimaging—whether it should be performed immediately or electively

The request of neuroimaging tests should follow a comprehensive process, involving the collection of anamnestic data, a thorough clinical examination, and the formulation of a well-defined diagnostic question. However, in real-world scenarios, especially with vertiginous patients, this general clinical approach is often disregarded. Notably, non-contrast cranial CT frequently substitutes clinical evaluation despite its limited sensitivity, serving as the primary examination for patients with vertigo [59, 60]. [refer to Supplementary Data “Diagnostic imaging”].

### Non-contrast computer tomography

Non-contrast computed tomography [NCCT] is the most commonly employed neuroimaging modality in ED for patients presenting with neurological symptoms, including acute vertigo. Despite its rapid accessibility [61–63], its diagnostic yield is limited, ranging from 2 to 10% [64, 65]. In cases of isolated acute vertigo without other indicative anamnestic or clinical findings of cerebral pathology, NCCT has been shown to lack additional diagnostic value. Recent American guidelines on isolated acute vertigo discourage its routine use [21]. Emergency department professionals should be aware of the test's low sensitivity, where a negative result may be misleading [10, 11] (Fig. 12).

### CT angiography

CT angiography [CTA] of the neck and intracranial vessels is essential in cases of suspected acute cerebral stroke or high-risk transient ischemic attack [66]. It should be performed immediately, preferably concurrently with the baseline cranial CT.

In the absence of suspected cerebrovascular pathology as the cause of vertiginous symptoms, CTA almost never contributes to the diagnostic process. This is supported by a retrospective study of 153 patients with nonspecific vertigo, where CTA was requested without a specific diagnostic question. Although the examination detected cerebral lesions in 5 patients, only 2 [2/153, 1.3%] had findings responsible for vertiginous symptoms [61], indicating a diagnostic yield of 1 in 100 examinations. This raises significant concerns regarding the cost–benefit ratio of the examination, particularly considering ionizing radiation exposure, renal damage, and contrast agent reactions, beyond economic considerations.

**Perfusion CT** [refer to Supplementary Data “Diagnostic imaging”].

### Magnetic resonance imaging

Magnetic Resonance Imaging [MRI] is the most sensitive imaging modality for detecting cerebral abnormalities and is considered the gold standard when suspecting lesions in the posterior cranial fossa. However, its disadvantages include limited availability in emergency departments, prolonged examination duration, and contraindications to execution.

In a review of five MRI studies encompassing 943 patients with acute vertigo [16, 63, 67, 68], the sensitivity for ischemic stroke was 79.8%, specificity was 98.8%, with no false positives identified [Fig. 13]. Nonetheless, a meta-analysis by Edlow et al. [8] indicates that in the case of small posterior circulation infarcts, a negative MRI with diffusion-weighted imaging [DWI] is five times more common than in anterior circulation infarcts. The identification of regions with diffusion restriction in the Diffusion-Weighted Imaging [DWI] sequence, indicative of acute ischemic areas, is time-dependent across all stroke cases [69]. Specifically, in instances of posterior circulation ischemia, the sensitivity of MRI significantly improves when conducted more than 48 h after symptom onset [70]. In patients with acute vestibular syndrome, MRI with DWI within the first 48 h fails to detect 10–22% of stroke cases [16, 72] and 50% of small posterior cranial fossa infarctions, half of which result from major vessel occlusion [71].

According to recent American guidelines [21], an MRI conducted within 48 h of symptom onset is less accurate than standardized clinical assessments, such as HINTS and STANDING, performed by appropriately trained emergency medicine specialists.

### Neuro-imaging in acute *vertigo* due to suspected cerebrovascular disease

In patients with acute vertigo from suspected cerebrovascular pathology (ischemic stroke, TIA, and intraparenchymal hemorrhage), it is crucial to promptly activate the stroke pathway to enable swift diagnostic confirmation, forming the basis for appropriate therapeutic measures. Clinical objectivity, incorporating the analysis of nystagmus, forms the basis for determining the timing and extent of required neuroimaging, categorizing patients into distinct groups:

1. Typical clinically peripheral forms: no neuroimaging tests are deemed necessary.
2. Non-typical peripheral and/or uncertain central forms lacking criteria for stroke pathway activation: urgent CT examination is not required, but elective MRI is recommended.
3. Clinical evaluation suggestive of a central form with criteria for stroke pathway activation, especially temporal criteria: emergency-urgent CT, CT angiography, and possibly deferred MRI examination (within 48–72 h) are warranted.
4. Unstable 'pseudo-dizziness' or those with urgent diagnostic suspicion (e.g., acute coronary syndrome, severe arrhythmia, aortic dissection): immediate attention is required for other urgent instrumental diagnostics.
5. Other non-urgent cases: other elective instrumental diagnostics should be considered as appropriate.

Regarding advanced imaging, such as Perfusion CT, refer to Supplementary Data “Diagnostic imaging”.

### Transient ischemic attack

While the traditional clinical definition of TIA hinges on the abrupt onset of neurological symptoms with complete resolution within 24 h, the American Heart Association, in 2009 [72], redefined TIA using a tissue-centric approach [resolution of symptoms and the absence of ischemic lesions on imaging]. Presently, TIA is characterized as an acute neurovascular syndrome linked to a swiftly resolving vascular territory, devoid of apparent ischemic lesions on MRI examination with DWI sequence. Conversely, patients displaying rapid symptom resolution with evident ischemic lesions on MRI are diagnosed with ischemic stroke.

**Fig. 7:**
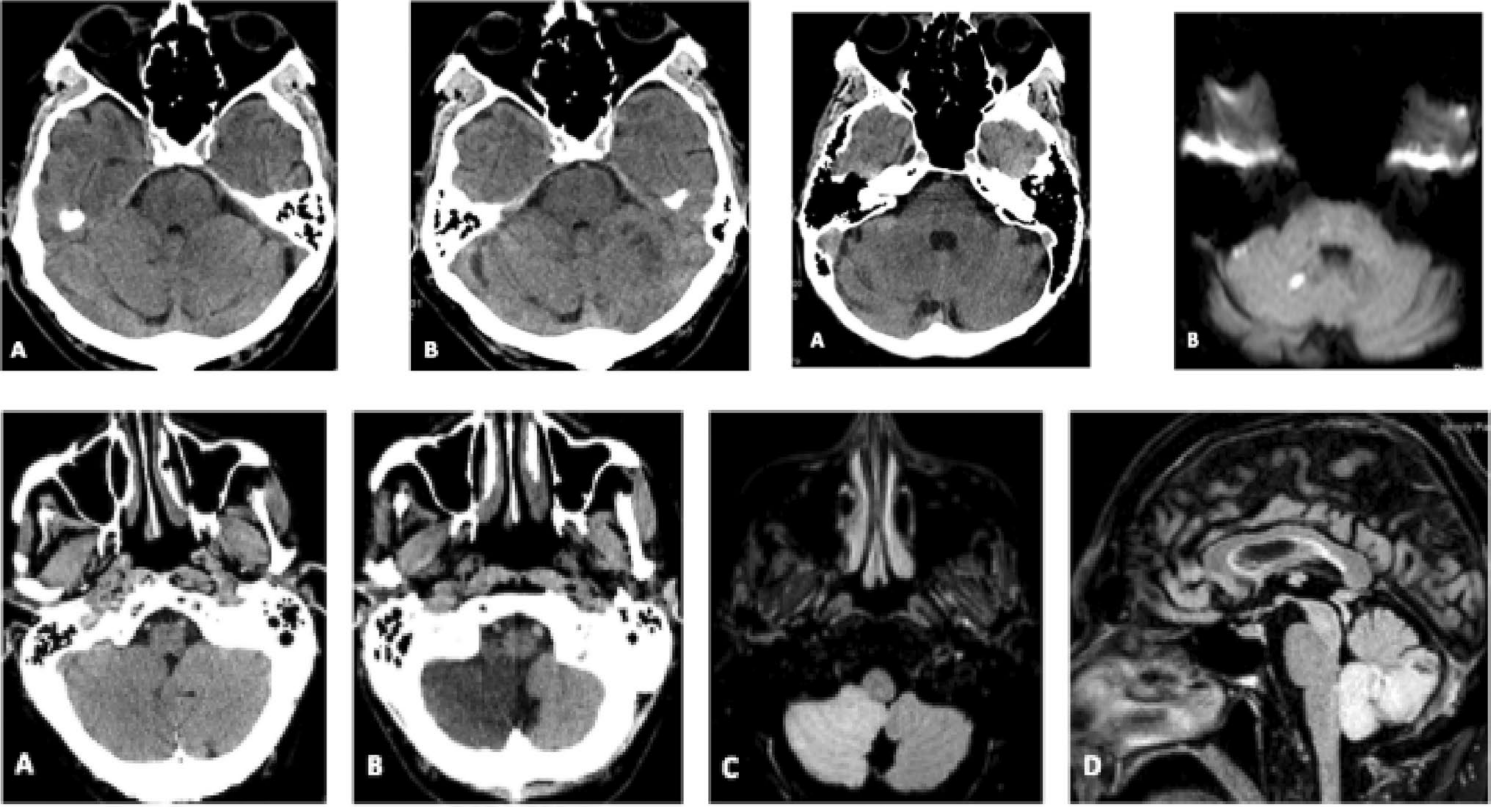
Upper panel: acute vertigo. **A** NCCT examination upon emergency department admission. **B** NCCT examination at 24 h from admission, revealing the emergence of a hypodense area indicative of acute vascular ischemia in the left cerebellar hemisphere. **C** NCCT examination upon emergency department admission. **D** MRI examination with Diffusion-Weighted Imaging [DWI] sequence at 72 h from admission, illustrating the presence of a diffusion restriction area suggestive of a recent vascular ischemic lesion in the right cerebellar hemisphere. Lower Panel: acute vertigo. **A** Baseline CT examination upon emergency department admission. **B** Baseline CT examination at 24 h from admission, demonstrating hypodensity in the right cerebellar hemisphere. **C** and **D** MRI examination with Fluid-Attenuated Inversion Recovery [FLAIR] sequence revealing hyperintensity in the territory of the right Posterior Inferior Cerebellar Artery [PICA]

In instances of suspected TIA, the neuro-imaging protocol should encompass baseline CT examination and CT angiography of the neck and intracranial vessels. Recent American Heart Association guidelines advocate for an MRI examination with a DWI sequence within 24 h of symptom onset [73]. However, this recommendation contrasts with other studies citing the risk of false negatives, especially in the initial 48 h, particularly in cases of posterior circulation ischemia [8, 68]. In clinical practice, the initial approach typically involves baseline CT examination combined with neck and intracranial vessel CT angiography, especially in high-risk TIA patients. Subsequently, in selected cases, an MRI examination with a delayed DWI sequence [> 48 h] is considered (Fig. 7).

## Treatment

### Benign paroxysmal positional *vertigo*

The treatment for Benign Paroxysmal Positional Vertigo [BPPV] is primarily physical, addressing its mechanical nature. The objective is to liberate the semicircular canal from otoconial debris, repositioning it into the utricle to facilitate reabsorption.

#### BPPV PCS therapy

The Semont maneuver [74] and the Epley repositioning maneuver [75] are the most commonly employed techniques for treating BPPV of the posterior semicircular canal [PSC] [refer to Supplementary Data “Benign Paroxysmal Positional Vertigo Treatment”].

**Fig. 8:**
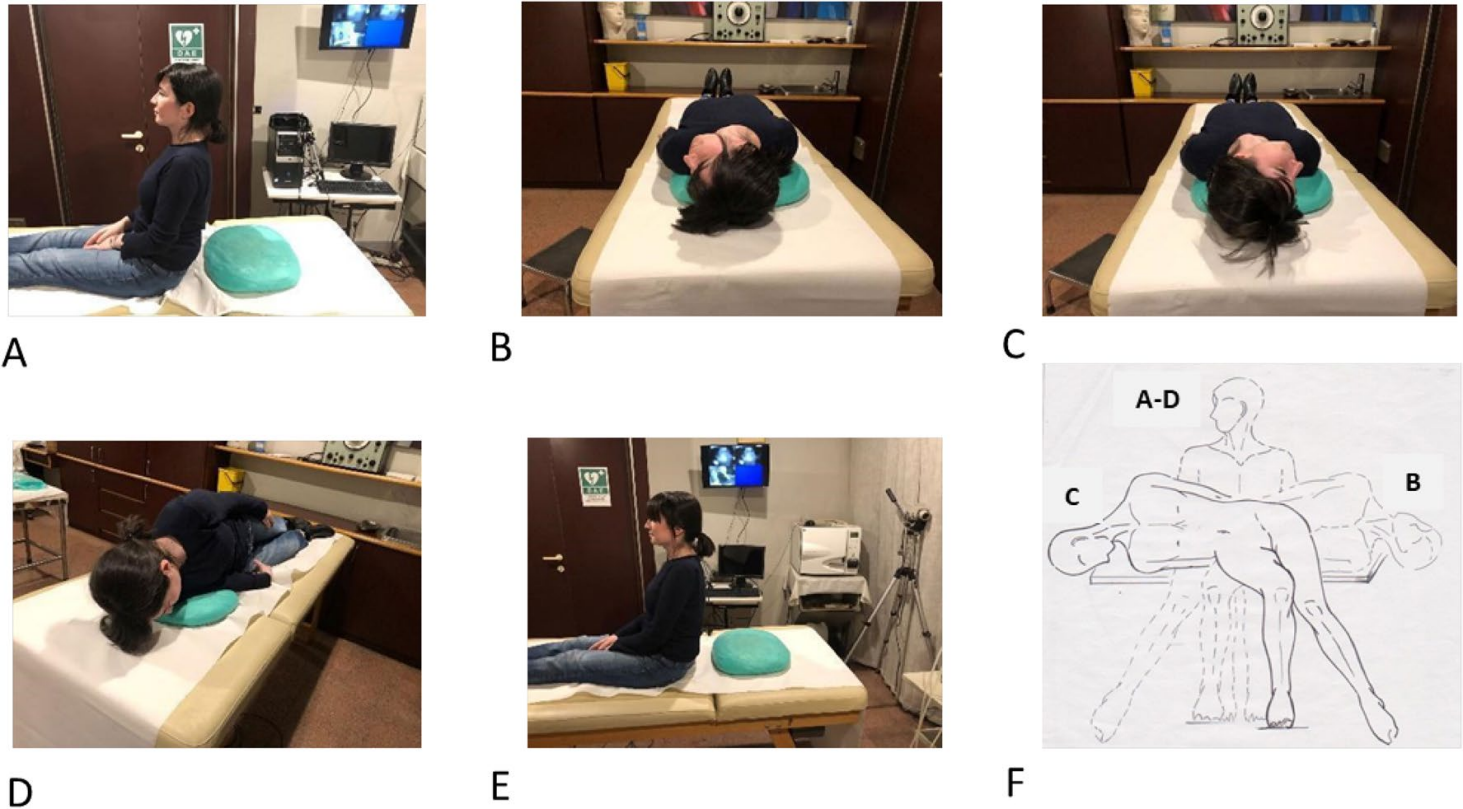
Epley Maneuver for BPPV of the left PSC **A** initiates the Epley maneuver from a seated position by turning the head 45° to the affected side [left] and progressing to the Dix-Hallpike left position. **B** Continue the rotation until reaching the Dix-Hallpike right position. **C** Subsequently, turn the patient until the face is directed downwards. **D** Conclude the maneuver by bringing the patient back to a sitting position [E]. Execute each step slowly, incorporating pauses of 30"—60". **F** Semont Maneuver for BPPV of the left PSC. Letters A, B, C, and D inside the Fig indicate the sequential phases of the Semont maneuver

Literature [76, 77] suggests that both liberating maneuvers are equally effective, resolving positional symptoms in one or two sessions. The choice between the Semont and Epley techniques depends primarily on the examiner's preference, patient mobility conditions, and, arguably, the characteristics of the nystagmus, particularly its intensity. If the nystagmus is not notably intense, indicating a lesser quantity and/or smaller dimensions of debris, the repositioning maneuver [Epley] may be more effective, leveraging the gravity of the debris, as opposed to the liberating maneuver [Semont], which relies on the inertia of the debris (Fig. 8).

#### BPPV LSC therapy

In the geotropic variant of LSC BPPV, two frequently employed techniques are the Gufoni maneuver [78] and the Vannucchi forced liberating position [79].

The Gufoni maneuver aims to dislodge otoconial debris from the LSC by leveraging a rapid deceleration. Initiated with the patient in a seated position, legs outside the bed, the maneuver swiftly transitions the patient to a lateral position on the side corresponding to the healthy side. [refer to Supplementary Data “Benign Paroxysmal Positional Vertigo Treatment”].

Vannucchi's forced liberating position necessitates the patient to maintain a lateral decubitus posture on the unaffected ear side for an extended period of 12 h, as reported in the original work. In this position, gravitational forces facilitate the movement of otoconial debris out of the affected canal towards the utricle. Due to its prolonged duration, this therapeutic approach is suitable for home treatment. At the time of diagnosis, the examiner educates the patient on the nature of their vertigo. Subsequently, the patient is advised to return home and assume a lateral decubitus posture corresponding to the healthy ear side. Nighttime is often recommended, as it aligns with the patient's sleep schedule. The examiner suggests the use of pillows to prevent inadvertent positional changes during sleep. Additionally, if the patient needs to rise, they are instructed to do so gradually, avoiding abrupt head movements, and to return to the lateral decubitus posture afterward.

When executed precisely and consistently, this maneuver boasts a symptom and sign resolution rate exceeding 90% within 24 h [79]. A modified version, involving a "shortened" forced liberating position lasting one hour, has been recently proposed. This adaptation serves to reduce the positioning duration while enabling real-time assessment of maneuver execution by the patient and the immediate efficacy of treatment. Approximately 70% of patients exhibited healing or significant improvement one hour after the abbreviated maneuver.

#### Drug therapy

Drug therapy plays a limited role in the management of BPPV; nevertheless, there are specific scenarios where pharmacological interventions can be beneficial. In patients exhibiting pronounced neurovegetative symptoms, the administration of antiemetic drugs such as metoclopramide, levosulpiride, or others may prove advantageous, allowing for the procedures to be conducted without excessive disruption.

Another circumstance warranting pharmacological consideration arises when a patient, engaged in physical therapy, experiences post-treatment instability in the subsequent days. This phenomenon is relatively common, and in such instances, the use of antihistamines or anticholinergics, such as dimenhydrinate or cinnarizine, can be highly beneficial. It is recommended to sustain this pharmacotherapy for a few days to address and manage the observed instability associated with physical therapy.

#### Acute peripheral vestibular dysfunction

The lack of clear understanding regarding the etiopathogenesis of APVD poses challenges in prescribing pharmacological interventions tailored to individual cases supported by robust scientific evidence. In practice, a substantial proportion of AVD patients, irrespective of the therapeutic approach employed, demonstrate gradual amelioration in rotatory vertigo within days following the acute episode. However, a notable subset, ranging from 20 to 50% of individuals, may exhibit persistent and consequential symptoms, including static and dynamic instability, and difficulty in maintaining steady visual fixation on an object [80].

The acute management of APVD involves the utilization of antiemetic and vestibule-suppressive medications to mitigate intense neurovegetative symptoms, such as nausea and vomiting, and reduce the severity of rotational vertigo. Vestibulo-suppressive drugs encompass anticholinergics, antihistamines [including cinnarizine, which exhibits anticholinergic and calcium channel-blocking properties], and benzodiazepines. Antiemetic drugs [e.g., metoclopramide, alizapride] are also valuable for alleviating neurovegetative symptoms, particularly when administered intravenously in the presence of pronounced emetic manifestations.

The administration of these drugs, at appropriate dosages, generally does not significantly alter the nystagmic profile. [refer to Supplementary Data “Acute Peripheral Vestibular Dysfunction Treatment”].

In light of the presumed viral etiology of APVD, the consideration of a brief corticosteroid regimen has been proposed [81], with the potential to enhance clinical outcomes in affected individuals [82]. While a recent meta-analysis concludes that the available evidence is insufficient to unequivocally support corticosteroid use in APVD patients [84], a retrospective study underscored that individuals treated with methylprednisolone [1 mg/kg for 5 days, followed by an additional 5 days at 0.5 mg/kg] exhibited superior outcomes in terms of vestibular signs and symptom recovery compared to those utilizing non-steroidal treatments. Despite these conflicting findings, it has been suggested that initiating steroid therapy within 24 h of APVD onset yields more favorable outcomes regarding the recovery of vestibular function in comparison to treatment initiated between 24 and 72 h post-onset [83].

### Ischemic stroke

#### Reperfusion therapy

For individuals experiencing recent-onset isolated vertigo, a critical challenge lies in discerning whether symptoms may be attributed to an acute cerebral ischemic event amenable to treatment. A review of randomized trials assessing fibrinolytic therapy in acute ischemic stroke underscores the efficacy of recombinant tissue plasminogen activator [rtPA] when administered systemically within 4.5 h of symptom onset and in the absence of contraindications to fibrinolysis. Results from the meta-analysis emphasize that the therapeutic benefit is more pronounced with earlier intervention, reinforcing the concept that treatment within the specified time frame is crucial [84].

Moreover, numerous randomized clinical trials establish that endovascular revascularization, encompassing mechanical thrombectomy and/or thrombo-aspiration within 6 h of symptom onset in patients with documented large vessel occlusion, leads to improved functional outcomes.

Practically, individuals arriving within 4.5 h of symptom onset should be promptly treated with rtPA. In cases where there is documented large vessel occlusion following systemic thrombolysis, mechanical thrombectomy is recommended. For patients presenting within the window of 4.5 to 6 h from symptom onset, mechanical thrombectomy should be considered if evidence of large vessel occlusion is present.

Advancements in neuroimaging techniques have expanded the treatment window for revascularization therapies, even in patients with symptoms persisting beyond 6 h. These sophisticated methods enable the identification of a favourable brain imaging profile, known as "mismatch," characterized by a substantial area of potentially salvageable ischemic penumbra relative to a smaller region of irreversible damage. Perfusion CT and MRI with diffusion and perfusion sequences, as detailed in the neuroimaging chapter, are these instrumental exams.

Utilizing these imaging methods, patients may be selected for systemic rtPA treatment between 4.5 and 9 h from symptom onset or in cases where the time of onset is undetermined, such as strokes occurring during sleep [85]. Applying the same neuroimaging criteria, various studies have reported positive outcomes for reperfusion treatments using endovascular techniques, extending the treatment window to up to 24 h from the onset of symptoms [86].

Concerning posterior circulation, a meta-analysis of randomized trials encompassing patients with acute stroke and basilar trunk occlusion underscores the superiority of mechanical thrombectomy over the best medical treatment. This superiority extends to reductions in both disability and mortality at the 90-day mark, albeit with an observed increase in symptomatic cerebral hemorrhages [87, 88]. Nevertheless, uncertainties persist regarding the practical application of this therapy in clinical settings.

Early secondary prophylaxis. [refer to Supplementary Data”Early secondary prophylaxis”].

## Proposal for a multi-professional pathway

### Triage and early evaluation

In the ED setting, patients presenting with dizziness or imbalance may access triage directly, utilizing independent pedestrian access, or arriving via emergency transportation managed by the Territorial Emergency Operations Center [TEOC]. The activation of TEOC services is initiated by dialing the universal emergency number, NUE 112, which serves as a single European point of contact for emergencies. The anamnestic approach, commencing from the pre-hospital activation phase of the emergency service, should mandate healthcare professionals to pose a specific set of standardized questions.

As a group of experts, we recommend incorporating the following key points into the triage interview:

- any prodromal symptoms or associated manifestations, especially those of a neurological or cardiovascular nature.
- triggering factors, specifically head or body movements, that might precipitate or exacerbate the vertigo.
- duration of the disturbance, whether it lasts for seconds, minutes, or hours.
- ability to maintain an upright position without significant difficulty.
- information concerning the patient's cardiovascular risk profile.

For further details refer to the “Medical History” chapter.

### Diagnostic assessment

**Fig. 9:**
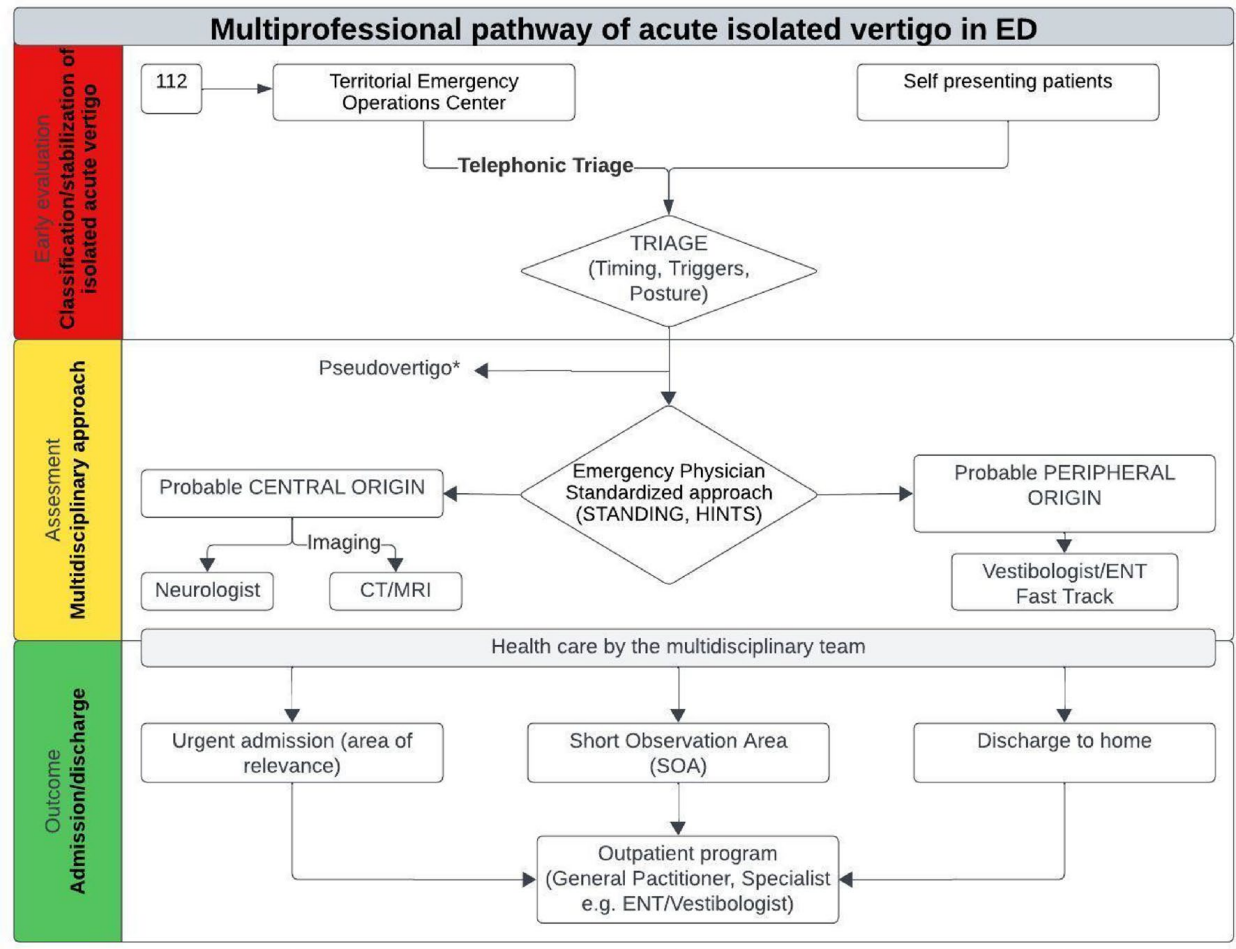
Multi-professional pathway of the patient with acute isolated vertigo in ED

The differentiation between peripheral vertigo and central vertigo, with a specific focus on identifying patients suspected of acute cerebrovascular pathology, is a critical juncture in decision-making. Subsequent to the initial triage phase, the implementation of a standardized approach, such as the STANDING algorithm, becomes pivotal. This can facilitate the early identification of the disorder in the post-triage or advanced triage operational area, where a multidisciplinary team comprising Medical Emergency Unit physicians and nurses, such as the Rapid Evaluation Team [TVR], is actively engaged (Fig. 9).

If the initial classification distinctly indicates a peripheral origin disorder, patients can be directed to an outpatient setting, thereby avoiding prolonged stays in the ED. Based on targeted anamnesis notes, suggestive of peripheral disorders such as BPPV, and a focused clinical evaluation, including the assessment of potential nystagmus [e.g., positional nystagmus] it is possible to orient towards a "discharge-fast-track " path in a hospital outpatient setting [typically, where it is available, with a 12-h daytime cycle]. This protocol is designed for prompt management and taking care of presumed isolated peripheral vertigo, facilitating timely care outside the urgent ED environment.

Diagnostic, clinical, and instrumental confirmations, along with the potential implementation of liberating maneuvers, are conducted in the outpatient setting by specialized personnel. This team will also initiate the planning of follow-up care, encompassing multidisciplinary hospital management. Any further investigations, such as non-urgent neuroimaging studies, will be coordinated through hospital pathways involving relevant specialists.

In the absence of causes of 'pseudovertigo' (Table 1), if clinical suspicion leans towards a central rather than peripheral pathology, further steps may involve the collaboration of other specialists, initiated based on a well-defined diagnostic inquiry.

For patients presenting with acute vertigo and suspected cerebrovascular involvement [including ischemic stroke, transient ischemic attack [TIA], and intraparenchymal hemorrhage], prompt activation of the stroke pathway is imperative to facilitate swift diagnostic confirmation. When selecting neuro-imaging tests, a crucial consideration is the distinction between 1st level centers [spoke], which are obligated to provide at least basic imaging following local protocols, and 2nd level centers [hub], where both basic and advanced imaging techniques, along with the requisite expertise, are available.

Clinical objectivity, incorporating the analysis of nystagmus, forms the basis for determining the timing and extent of required neuroimaging (see chapter “Diagnostic Imaging”, page 23).

### The exit from the ED: discharge, observation or hospitalization

One distinctive aspect of the emergency physician lies in discerning between patients necessitating hospitalization and those suitable for discharge. The patient can be assisted taking into account the complexity of care and the diagnostics to be performed according to a specific time criterion by priority. Collaboration among various professionals, including nurses, emergency physicians, vestibologist, neurologists, and radiologist, is fundamental in formulating and executing the instrumental diagnostic and care pathway. This pathway takes into consideration:

- Care complexity
- Specialist relevance
- Prescriptive appropriateness
- Diagnostic priority
- Adherence to timeline
- Final transition of care

When the patient does not need immediate diagnostic procedures, discharge can be directly facilitated from ED to the relevant specialty service overseeing the case. Conversely, if diagnostic tests are mandated during the hospitalization phase, the specialized team managing the case will coordinate the planning and/or execution of requisite tests, each in accordance with their respective areas of expertise.

In instances where deferrable diagnostics are deemed necessary in the outpatient setting, it is advisable to delineate, at the local level, both the timing and methodologies for the required tests. Furthermore, a designated specialist responsible for the ultimate patient management should be identified. The patient will then undergo remote re-evaluation [e.g., outpatient clinic/Day Service] for diagnostic conclusions and therapeutic recommendations.

### The short observation area

The Short Observation Area of the ED *is designed to accommodate patients with isolated vertigo when there is a well-defined diagnostic and therapeutic plan from the outset*. The primary objectives include confirming a diagnostic hypothesis (central vertigo), stabilizing a clinical condition, and correcting a pathological framework. This is achieved through a collaboratively planned approach, involving agreed-upon investigations and monitoring, adhering to the anticipated duration of stay in the area (36–48 h).

A precise definition of the vertigo management pathway is imperative to ensure adherence to key system indicators such as prescription appropriateness, duration of stay in the SOA, and prevention of inappropriate hospital admissions.

## Perspectives and limitations

Vertigo is a common issue in the ED. However, in a notable subset of patients, it may be indicative of a central nervous system pathology necessitating urgent intervention. Brain CT utilization in these cases demonstrates low sensitivity, while clinical evaluation by experts or validated simplified algorithms has proven more effective for accurate differential diagnosis. Unfortunately, many EDs lack access to highly specialized professionals like neuro-otologists or vestibologists, 24/24 h and 7/7 days.

Despite single-center studies indicating that emergency physicians often inadequately perform nystagmus assessments, diagnostic maneuvers, or misapply them, conflicting evidence from other studies exists [5, 20, 57]. The primary limitation in correctly classifying patients with isolated vertigo appears to be the absence of sufficient specialized training.

It is crucial to develop specific training tools and widely disseminate them rapidly. This effort aims to ensure that an increasing number of emergency doctors can adeptly employ and interpret the fundamental and simplest clinical tests.

We recommend that training in this context primarily be based on theoretical-practical courses enriched with demonstration videos illustrating various types of nystagmus and essential diagnostic maneuvers.

While there is limited data on the duration and number of tutored exams required to achieve adequate practical competence, preliminary studies suggest that a few hours of lectures accompanied by a limited number of practical tests are sufficient for attaining a competent level [5, 20, 57].

Incorporating more modern methods for detecting eye movements, such as video-oculography, into daily practice could facilitate interaction, consultation, and feedback from experts through telemedicine systems. Notably, there is a notable absence of large multicenter studies substantiating the application of both HINTS and STANDING protocols in ED settings.

## Conclusions

The progress achieved thus far underscores the critical importance of open and constructive communication among diverse specialists. Vertigo, a symptom involving multiple specialties, demands observation, evaluation, and framing from various perspectives.

This consensus document, a collaborative effort between the Italian Society of Emergency Medicine [SIMEU] and the Italian Society of Vestibology [VIS], in conjunction with neurologists and neuroradiologists, represents an initial stride at the national level. Its purpose is to lay the foundational groundwork essential for constructing accurate and shared clinical pathways.

It is crucial to emphasize that, without a tangible commitment to widespread dissemination and training, facilitated through scientific societies and other institutional [university and national health service] channels, the recommendations in this document remain aspirational rather than established benchmarks for good clinical practice.

### Supplementary Information

Below is the link to the electronic supplementary material.

Acute Peripheral Vestibular Dysfunction (DOCX 171 KB)

Additional Diagnostic Maneuvers (DOCX 20 KB)

Benign Paroxysmal Positional Vertigo (DOCX 1107 KB)

Diagnostic algorithm (DOCX 21 KB)

Diagnostic imaging (DOCX 27 KB)

Early secondary prophylaxis (DOCX 17 KB)

Focused physical examination (DOCX 21 KB)

Medical History (DOCX 24 KB)

Methods (DOCX 16 KB)

Nystagmus Patterns of Central Origin (DOCX 17 KB)

The clinical examination of ocular motility (DOCX 18 KB)

## Data Availability

Considering that the manuscript does not concern a clinical study, there are no data to provide.

## Abbreviations

**ACS**: Anterior semicircular canal
**AICA**: Anterior inferior cerebellar artery
**APVD**: Acute peripheral vestibular dysfunction
**AVrS**: Acute vertiginous syndrome
**BPPV**: Benign paroxysmal positional vertigo
**CNS**: Central nervous system
**CPPV**: Central paroxysmal positional vertigo
**CT**: Computed tomography
**CTP**: Perfusion computed tomography
**DWI-MR**: Diffusion-weighted imaging-magnetic resonance
**ED**: Emergency department
**EEG**: Electroencephalogram
**EKG**: Electrocardiogram
**EVrS**: Episodic vertiginous syndrome
**HINTS**: Head Impulse test, Nystagmus, Test of Skew
**HIT**: Head impulse test
**HST**: Head shaking test
**LSC**: Lateral semicircular canal
**MD**: Ménière disease
**NCCT**: Non-contrast computed tomography
**NIHSS**: National Institutes of Health Stroke Scale
**NMR**: Nuclear magnetic resonance
**OKR**: Opto-kinetic reflex
**OLD**: Ocular lateral deviation
**OTR**: Ocular tilt reaction
**PAN**: Periodic alternating nystagmus
**PICA**: Posterior inferior cerebellar artery
**PSC**: Posterior semicircular canal
**RAT**: Rapid assessment team
**RCT**: Randomized controlled trial
**rtPA**: Recombinant tissue plasminogen activator
**SCA**: Superior cerebellar artery
**s-EVrS**: Spontaneous episodic vertiginous syndrome
**SIMEU**: Società Italiana di Medicina di Emergenza Urgenza
**SP**: Smooth pursuit
**t-EVrS**: Triggered episodic vertiginous syndrome
**TIA**: Transient ischemic attack
**VIS**: Vestibology Italian Society
**VM**: Vestibular migraine
**VN**: Vestibular neuritis
**VOR**: Vestibulo ocular reflex
**VOS**: Video-oculo-scopy
**VSR**: Vestibular spinal reflex
**WG**: Working group
