# Supplementary material for: Consensus paper on the management of acute isolated vertigo in the emergency department
Source: Intern Emerg Med. 2024 Jul 13;19(5):1181–202. doi: 10.1007/s11739-024-03664-x (PMC11364714; doi:10.1007/s11739-024-03664-x)
Supplement: Supplementary file 1 — Acute Peripheral Vestibular Dysfunction (DOCX 171 KB) [file 11739_2024_3664_MOESM1_ESM.docx]

**Acute Peripheral Vestibular Dysfunction**

**Epidemiology**

While Ruttin [1] provided the initial description of APVD in the literature, Nylen [2] and Dix and Hallpike [3] were among the first to use the term VN, aiming to distinguish it from MD. It was Lindsay and Hemenway [4], and subsequently Fetter and Dichgans [5], who advocated for the more accurate and generic term APVD. This terminology, "APVD," is the one adopted in this document.

As the diagnostic criteria for APVD have lacked strict uniformity over the years, there are currently no epidemiological studies adequately addressing this question. Some propose APVD as the most common cause of prolonged spontaneous acute vertigo, constituting 3.2-9% of visits to specialized centers for balance disorders [6-7]. Others suggest an annual incidence of 3.5-15.5% cases per 100,000 individuals [8-9]. The annual incidence rate of APVD in Italy has recently been estimated to be between 18-20/100,000 [10].

**Clinical features of nystagmus**

The nystagmus is *persistent*, enduring as long as it is observed, and maintains a *stable* character over time, with its speed remaining essentially unchanged. In the acute phase, fixation inhibition may be only partial [observable even under fixation].Before attributing nystagmus with reasonable certainty to a labyrinthine origin, indicative of unilateral APVD, the Head Impulse Test [HIT] should be conducted [11]. This test verifies the absent or reduced function of VOR on the affected side. A positive result after a rapid [impulsive] rotation towards the affected side, opposite to the direction of spontaneous nystagmus, suggests that the tested labyrinth is incapable of generating an effective VOR. Consequently, it is highly probable that the patient has a problem of peripheral vestibular nature [12].  An expression of utricle damage, evident only in the early phases of APVD, is the Ocular Tilt Reaction [OTR]. This encompasses cyclotorsion of the eyes, head tilt towards the pathological side, and slight vertical misalignment of the two eye globes. However, these findings, challenging to evaluate in the acute phase due to the overlaid violent spontaneous nystagmus, undergo early exhaustion [within hours or days] due to central compensatory mechanisms [13-14] [Fig 1].

The assessment of the combination of spontaneous nystagmus and OTR can sometimes aid in attributing a judgment regarding the site of pathology causing the nystagmus. It has been proposed that an "uphill" nystagmus [beating towards the hypertropic eye] suggests a peripheral origin, while a "downhill" nystagmus [directed towards the hypotropic eye] should be evaluated for a possible central genesis [15].


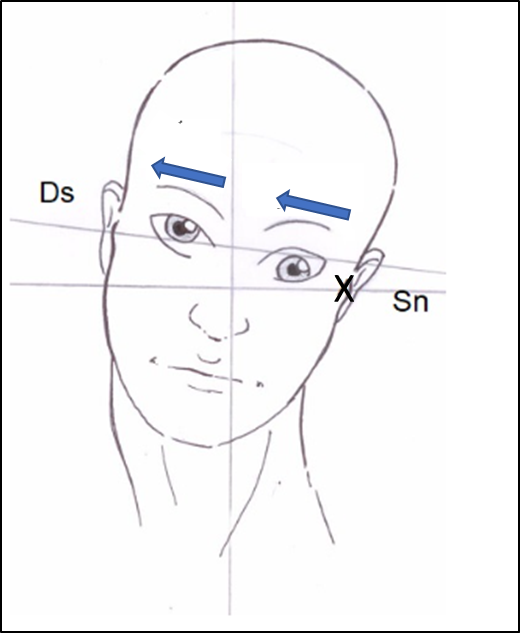


**Fig 1.** Representation of Ocular Tilt Reaction [OTR]. In a patient with left-sided Acute Peripheral Vestibular Dysfunction [APVD], the head tilts towards the left, the left eye is positioned lower [hypotropic], and the right eye tends to be higher [hypertropic]. Simultaneously, the nystagmus, as indicated by the arrows, beats towards the right ear, so-called ‘uphill nystagmus’.

**Treatment**

Some medications exhibit dual effects; for instance, dimenhydrinate acts as both an H1 antagonist and possesses anticholinergic properties, leading to equivalent efficacy compared to diazepam. In the acute phase, intravenous dimenhydrinate has demonstrated superior efficacy over intravenous lorazepam [16]. Additionally, the combined use of cinnarizine and dimenhydrinate has shown heightened effectiveness in reducing vertigo and neurovegetative symptoms, surpassing even the efficacy of betahistine treatment [17,18]. Concerning betahistine, a partial agonist of H1 receptors and a potent antagonist of H3 receptors inducing an elevation in histamine release and turnover, there is some evidence supporting a positive impact on symptoms of dizziness [19]. However, the methodological heterogeneity and imprecise patient selection in clinical trials hinder a definitive determination of the true efficacy of betahistine APVD. While experimental animal studies demonstrate betahistine's effectiveness in promoting vestibular compensation post-APVD [20], its impact seems more apparent in the phase after the acute episode [21].

Regardless, the use of vestibular suppressant therapy, including betahistine, should be limited to a brief duration [2-5 days] to prevent potential adverse effects on the vestibular compensation process [22]. Simultaneously, patients should be actively encouraged to swiftly regain mobility and increase physical activity as soon as it becomes tolerable. Notably, expedited engagement in early active mobilization accelerates functional recovery more effectively [23].

**References**

1. Ruttin E. Zur Differentialdiagnose der Labyrinth- und Hörnerverkrankungen. *Z Ohrenheilkunde* 1909;57:327–333.
2. Nylen CO. Some cases of ocular nystagmus due to certain positions of the head. *Acta Otolaryngol [Stockh]* 1924;6:106–137
3. Dix MR, Hallpike CS. The Pathology, Symptomatology and Diagnosis of Certain Common Disorders of the Vestibular System. Proc R Soc Med. 1952
4. Lindsay JR, Hemenway WG. Postural vertigo due to unilateral sudden partial loss of vestibular function. *Arch Otolaryngol* 1956;65:692–706.
5. Fetter M, Dichgans J. Three-dimensional human VOR in acute vestibular lesions. *Ann N Y Acad Sci* 1996;781:619–621
6. Kroenke K, Hoffman RM, Einstadter D. How common are various causes of dizziness? A critical review. *South Med J*. 2000;93[2]:160-168.
7. Strupp M, Brandt T. Diagnosis and treatment of vertigo and dizziness. *Dtsch Arztebl Int*. 2008 Mar;105[10]:173-80
8. Adamec I, Krbot SM, Handzic J, Habek M. Incidence, seasonality and comorbidity in vestibular neuritis. *Neurol Sci* 2015;36:91–95.
9. Sekitani T, Imate Y, Noguchi T, Inokuma T. Vestibular neuronitis: epidemiological survey by questionnaire in Japan. *Acta Otolaryngol Suppl*. 1993;503:9-12
10. Ma[rco Mandalà](https://www.frontiersin.org/people/u/19939). The incidence of vestibular neuritis in Italy. Front. Neurol. 2023
11. Halmagyi GM, Curthoys IS. A clinical sign of canal paresis. *Arch Neurol* 1988;45:737-773.
12. Strupp M, Bisdorff A, Furman J, et al. Acute unilateral vestibulopathy/vestibular neuritis: Diagnostic criteria. *J Vestib Res*. 2022;32[5]:389-406.
13. Brandt T. Stroke and vertigo. In: Brandt T, editor. Vertigo: Its multisensory syndrome 2nd ed. London: *Springer-Verlag*; 2003. p. 307–24.
14. Guidetti G, Comacchio F, Casani A.P. Trattato italiano di vestibologia clinica, *Cleup,* 2021.
15. Gufoni M. [Uphill/downhill nystagmus.](https://pubmed.ncbi.nlm.nih.gov/29327737/) *Acta Otorhinolaryngol Ital*. 2017;37[6]:513-518
16. Keith A. Marill MD, Matthew J. Walsh MD, Brian K. Nelson MD, MS Intravenous lorazepam versus dimenhydrinate for treatment of vertigo in the emergency department: A randomized clinical trial. Academic Emergency Medicine annual meeting, San Francisco, CA, May 2000.
17. [Ales Hahn](https://pubmed.ncbi.nlm.nih.gov/?sort=pubdate&term=Hahn+A&cauthor_id=18211117), [Ivan Sejna](https://pubmed.ncbi.nlm.nih.gov/?sort=pubdate&term=Sejna+I&cauthor_id=18211117), [Bohdana Stefflova](https://pubmed.ncbi.nlm.nih.gov/?sort=pubdate&term=Stefflova+B&cauthor_id=18211117), [Mario Schwarz](https://pubmed.ncbi.nlm.nih.gov/?sort=pubdate&term=Schwarz+M&cauthor_id=18211117), [Wolfgang Baumann](https://pubmed.ncbi.nlm.nih.gov/?sort=pubdate&term=Baumann+W&cauthor_id=18211117). A fixed combination of cinnarizine/dimenhydrinate for the treatment of patients with acute vertigo due to vestibular disorders : a randomized, reference-controlled clinical study. Clin Drug Investig. 2008;28[2]:89-99.
18. [Arne-Wulf Scholtz](https://pubmed.ncbi.nlm.nih.gov/?sort=pubdate&term=Scholtz+AW&cauthor_id=22506537), [Raluca Steindl](https://pubmed.ncbi.nlm.nih.gov/?sort=pubdate&term=Steindl+R&cauthor_id=22506537), [Nicole Burchardi](https://pubmed.ncbi.nlm.nih.gov/?sort=pubdate&term=Burchardi+N&cauthor_id=22506537), [Irene Bognar-Steinberg](https://pubmed.ncbi.nlm.nih.gov/?sort=pubdate&term=Bognar-Steinberg+I&cauthor_id=22506537), [Wolfgang Baumann](https://pubmed.ncbi.nlm.nih.gov/?sort=pubdate&term=Baumann+W&cauthor_id=22506537) Comparison of the therapeutic efficacy of a fixed low-dose combination of cinnarizine and dimenhydrinate with betahistine in vestibular neuritis: a randomized, double-blind, non-inferiority study. Clin Drug Investig. 2012 Jun 1;32[6]:387-99
19. [Jozef J P Nauta](https://pubmed.ncbi.nlm.nih.gov/?sort=pubdate&term=Nauta+JJ&cauthor_id=23778722). Meta-analysis of clinical studies with betahistine in Ménière's disease and vestibular vertigo. Eur Arch Otorhinolaryngol. 2014 May;271[5]:887-97.
20. B. Tighilet et al. [Histaminergic ligands improve vestibular compensation in the cat: behavioral, neurochemical and molecular evidence](https://www.sciencedirect.com/science/article/pii/S0014299907005316). Eur J Pharmacol. 2007
21. [Kazunori Matsuda](https://pubmed.ncbi.nlm.nih.gov/?sort=pubdate&term=Matsuda+K&cauthor_id=30990106), [Tadashi Kitahara](https://pubmed.ncbi.nlm.nih.gov/?sort=pubdate&term=Kitahara+T&cauthor_id=30990106), [Taeko Ito](https://pubmed.ncbi.nlm.nih.gov/?sort=pubdate&term=Ito+T&cauthor_id=30990106), [Munehisa Fukushima](https://pubmed.ncbi.nlm.nih.gov/?sort=pubdate&term=Fukushima+M&cauthor_id=30990106), [Junya Fukuda](https://pubmed.ncbi.nlm.nih.gov/?sort=pubdate&term=Fukuda+J&cauthor_id=30990106), [Go Sato](https://pubmed.ncbi.nlm.nih.gov/?sort=pubdate&term=Sato+G&cauthor_id=30990106), [Yoshiaki Kitamura](https://pubmed.ncbi.nlm.nih.gov/?sort=pubdate&term=Kitamura+Y&cauthor_id=30990106), [Koji Abe](https://pubmed.ncbi.nlm.nih.gov/?sort=pubdate&term=Abe+K&cauthor_id=30990106), [Atsuhiko Uno](https://pubmed.ncbi.nlm.nih.gov/?sort=pubdate&term=Uno+A&cauthor_id=30990106), [Koichi Tomita](https://pubmed.ncbi.nlm.nih.gov/?sort=pubdate&term=Tomita+K&cauthor_id=30990106), [Hiromi Sakata-Haga](https://pubmed.ncbi.nlm.nih.gov/?sort=pubdate&term=Sakata-Haga+H&cauthor_id=30990106), [Yoshihiro Fukui](https://pubmed.ncbi.nlm.nih.gov/?sort=pubdate&term=Fukui+Y&cauthor_id=30990106), [Noriaki Takeda](https://pubmed.ncbi.nlm.nih.gov/?sort=pubdate&term=Takeda+N&cauthor_id=30990106). A new immunohistochemical method to evaluate the development of vestibular compensation after unilateral labyrinthectomy in rats. Acta Otolaryngol. 2019 Jun;139[6]:505-510.
22. [Augusto Pietro Casani](https://pubmed.ncbi.nlm.nih.gov/?term=Casani%20%20AP%5BAuthor%5D), [Mauro Gufoni](https://pubmed.ncbi.nlm.nih.gov/?term=Gufoni%20M%5BAuthor%5D), [Silvia Capobianco](https://pubmed.ncbi.nlm.nih.gov/?term=Capobianco%20S%5BAuthor%5D). Current Insights into Treating Vertigo in Older Adults. [Drugs Aging.](https://www.ncbi.nlm.nih.gov/pmc/articles/PMC8342368/) 2021; 38[8]: 655–670.
23. [Courtney D Hall](https://pubmed.ncbi.nlm.nih.gov/?sort=pubdate&term=Hall+CD&cauthor_id=26913496), [Susan J Herdman](https://pubmed.ncbi.nlm.nih.gov/?sort=pubdate&term=Herdman+SJ&cauthor_id=26913496), [Susan L Whitney](https://pubmed.ncbi.nlm.nih.gov/?sort=pubdate&term=Whitney+SL&cauthor_id=26913496), [Stephen P Cass](https://pubmed.ncbi.nlm.nih.gov/?sort=pubdate&term=Cass+SP&cauthor_id=26913496), [Richard A Clendaniel](https://pubmed.ncbi.nlm.nih.gov/?sort=pubdate&term=Clendaniel+RA&cauthor_id=26913496), [Terry D Fife](https://pubmed.ncbi.nlm.nih.gov/?sort=pubdate&term=Fife+TD&cauthor_id=26913496), [Joseph M Furman](https://pubmed.ncbi.nlm.nih.gov/?sort=pubdate&term=Furman+JM&cauthor_id=26913496), [Thomas S D Getchius](https://pubmed.ncbi.nlm.nih.gov/?sort=pubdate&term=Getchius+TS&cauthor_id=26913496), [Joel A Goebel](https://pubmed.ncbi.nlm.nih.gov/?sort=pubdate&term=Goebel+JA&cauthor_id=26913496), [Neil T Shepard](https://pubmed.ncbi.nlm.nih.gov/?sort=pubdate&term=Shepard+NT&cauthor_id=26913496), [Sheelah N Woodhouse](https://pubmed.ncbi.nlm.nih.gov/?sort=pubdate&term=Woodhouse+SN&cauthor_id=26913496). Vestibular Rehabilitation for Peripheral Vestibular Hypofunction: An Evidence-Based Clinical Practice Guideline: FROM THE AMERICAN PHYSICAL THERAPY ASSOCIATION NEUROLOGY SECTION. J Neurol Phys Ther. 2016 Apr;40[2]:124-55.7
