# Supplementary material for: Consensus paper on the management of acute isolated vertigo in the emergency department
Source: Intern Emerg Med. 2024 Jul 13;19(5):1181–202. doi: 10.1007/s11739-024-03664-x (PMC11364714; doi:10.1007/s11739-024-03664-x)
Supplement: Supplementary file 2 — Additional Diagnostic Maneuvers (DOCX 20 KB) [file 11739_2024_3664_MOESM2_ESM.docx]

**Additional Diagnostic Maneuvers**

*Assessment of the Glossopharyngeal Nerve (Cranial Nerve IX)*

The role of the glossopharyngeal nerve is to enable adequate and functional swallowing. A lesion of the cranial nerve IX (rarely isolated) can lead to taste loss in the posterior third of the tongue, hypoesthesia of the tonsil, uvula, and pharynx, difficulty in swallowing, and unilateral abolition of the gag reflex (or pharyngeal). To assess the function of the glossopharyngeal nerve, the posterior wall of the pharynx can be stimulated with a cotton swab or a tongue depressor causing, under physiological conditions, a contraction of the superior pharyngeal constrictor muscle (which tightens and elevates the posterior pharyngeal wall), sometimes associated with attempts to vomit (pharyngeal reflex) (along with the vagus nerve). Additionally, the subject can be asked to open their mouth, keeping the tongue lowered, and produce a sharp sound corresponding to the vowel "A": if facing unilateral paralysis of the IX cranial nerve, the posterior wall on the damaged side would move towards the healthy side, generating the so-called "Curtain Sign" (the uvula moves towards the healthy side). Finally, by evaluating swallowing for liquids and solids, it is possible to assess the integrity of the IX (and X) cranial nerve, as this is responsible for adequate contraction of the superior pharyngeal constrictor and the closure of the epiglottis.
 It is important to underline that the glossopharyngeal nerve and the vagus nerve (X) are deeply interconnected both functionally and anatomically.

*Assessment of the Vagus nerve (Cranial Nerve X)*

The vagus nerve is the tenth cranial nerve. It consists of a motor part (for the larynx and pharyngeal constrictor muscles), a sensory part (for the meninges of the posterior cranial fossa, the outer ear, and taste impulses from the epiglottis), and a substantial vegetative part (for the control of the heart, trachea, bronchi, and digestive tract). The vagus nerve can be investigated in its visceral motor component and in its somatosensory cutaneous component, as the vegetative part is difficult to assess. It is possible to assess the muscles of the soft palate as described above for the glossopharyngeal nerve. A lesion of the cranial nerve X typically can lead to ipsilateral paralysis of the soft palate, i.e. hypophonia and rhinolalia, ineffective swallowing, asymmetric uvula with deviation towards the healthy side, and a sluggish response to the pharyngeal reflex. It also causes unilateral paralysis of the larynx (of one vocal cord) and the "curtain sign" may be present. Moreover, there may be greater difficulty swallowing liquids and semisolids compared to solids. Furthermore, pathology affecting the vagus nerve could cause cardiac, respiratory, and digestive problems. Indeed, the vagus nerve influences the proper functioning of organs and can affect the parasympathetic nervous system.

*Bernard-Horner syndrome*

The Bernard-Horner syndrome is due to a deficit of the cervical sympathetic system and it is characterized by eyelid ptosis, ipsilateral miosis, enophthalmos and sometimes facial anhidrosis. In Bernard-Horner syndrome anisocoria may sometimes only be evident in dim light when the normal eye tends towards mydriasis due to the lack of light. This syndrome can occur because of a lesion along the entire course of the cervical sympathetic, including a stroke involving the vestibular nuclei or the inferior cerebellar peduncle at the level of the medulla oblongata.

*Head Shaking Test*

During this examination, the patient is seated while donning Frenzel glasses or a Video-Oculo-Scopy [VOS] mask. The procedure involves rotating the patient's head, inducing 20-30 oscillations of medium amplitude at a frequency of 1-2 shocks per second. Following the shaking, the patient is instructed to gaze straight ahead. In a healthy individual, no nystagmus should be present.

The emergence of nystagmus, referred to as "head-shaking nystagmus," is indicative of a peripheral pathology, often manifested as horizontal nystagmus beating towards the healthy labyrinth. Notably, the appearance of a downward vertical nystagmus subsequent to a Head-Shaking Test [HST] performed on the horizontal plane, known as "perverted head shaking nystagmus," raises suspicion for a central type of vertigo.

*‘Ocular lateral deviation’*

This straightforward and rapid diagnostic maneuver serves as a valuable tool for distinguishing peripheral from central AVS. The patient is instructed to fixate on a point approximately one meter away without moving their head. In the central position, the patient gently closes their eyes for 30-35 seconds, followed by reopening their eyes and fixing the target once again. Upon the patient's eye reopening, careful observation is made for the return of their gaze from the extreme lateral position to the median position. Although relatively uncommon in patients with AVrS [8.4% of cases], this sign demonstrates high specificity for central dysfunction and is typically suggestive of a lateral infarction of the bulb [1].

*The hyperventilation test*

During this diagnostic procedure, the patient is directed to hyperventilate for a duration of 60-70 seconds, and the subsequent appearance of nystagmus is carefully observed. Hyperventilation induces hypocapnia, leading to hypoxia and cellular alkalosis, consequently increasing neuronal excitability. In a normal subject, this test typically does not elicit the appearance of nystagmus, resulting in few false positives. In the case of APVD, the hyperventilation test may yield positive results in both excitatory [increases the intensity of the nystagmus] and inhibitory [the nystagmus is reduced] senses, depending on the compensation phases [2,3].

*Evaluation of standing and walking*

The act of standing up and walking is a fundamental and indispensable component of assessing a patient with vertigo. If the patient exhibits an inability to walk without support or struggles to stand unaided, it is imperative to consider the presence of a central pathology [4].

**References**

1. Kattah JC, Badihian S, Pula JH, Tarnutzer AA, Newman-Toker DE, Zee DE. *Ocular lateral deviation with brief removal of visual fixation differentiates central from peripheral vestibular syndrome.* J Neurol. 2020 December ; 267[12]: 3763–3772.
2. Califano L, Melillo MG, Vassallo A, Mazzone S. Hyperventilation.induced nystagmus in a large series of vestibular patients. Acta Otorhinolaringol Ital. 2011; 31, 17-26.
3. Califano L, Locatelli G, Melillo MG. Can hyperventilation test and duration of spontaneous nystagmus help differentiate between vascular and inflammatory aetiology of acute unilateral vestibular deficit? Acta Otorhinolaryngol Ital. 2022;42[6]:560-568
4. Carmona S, Martínez C, Zalazar G, Moro M, Batuecas-Caletrio A, Luis L, Gordon C. The Diagnostic Accuracy of Truncal Ataxia and HINTS as Cardinal Signs for Acute Vestibular Syndrome. Front Neurol. 2016 Aug 8;7:125.
