# Supplementary material for: Consensus paper on the management of acute isolated vertigo in the emergency department
Source: Intern Emerg Med. 2024 Jul 13;19(5):1181–202. doi: 10.1007/s11739-024-03664-x (PMC11364714; doi:10.1007/s11739-024-03664-x)
Supplement: Supplementary file 3 — Benign Paroxysmal Positional Vertigo (DOCX 1107 KB) [file 11739_2024_3664_MOESM3_ESM.docx]

**Benign Paroxysmal Positional Vertigo**

**Clinical assessment**

**BPPV-PSC**

During the Dix-Hallpike manoeuvre [Fig.3 of Main Manuscript], the excitatory stimulus induces an upward rotational/vertical nystagmus [rotatory-up]. This results from the contraction of the ipsilateral superior oblique muscle and the contralateral inferior rectus. The rapid phase of the nystagmus is directed towards the right shoulder [counterclockwise-up for the observer] for right-sided stimulation and towards the left shoulder [clockwise-up for the viewer] for left-sided stimulation. A latency period is often observed, likely attributed to material overcoming resistance within the ampoule and/or canal before movement occurs. The nystagmus is paroxysmal, intensifying rapidly, reaching a peak within seconds, and then diminishing. Returning the patient to a sitting position induces an endolymphatic current in the opposite direction, resulting in a nystagmus mirroring the hyperextended head position but of lower intensity due to its inhibitory nature.

Repeated positioning maneuvers may elicit recurrent nystagmus, which tends to be fatigable, gradually diminishing in intensity until it may disappear. The observation of this nystagmus, in conjunction with accompanying symptoms, allows for a definitive diagnosis of BPPV-PSC. When executed correctly, the maneuver reveals a clear and recognizable nystagmus pattern, often exhibiting similarities among different patients, thereby facilitating accurate diagnosis with experience.

**BPPV-LSC**

In certain instances, nystagmus directions may be reversed, such as a right nystagmus on the left side and vice versa. This occurrence is characteristic of the apogeotropic variant of BPPV involving the lateral semicircular canal [BPPV-LSC] [for further details, refer to online materials]. Geotropic forms are often more uniform and easily recognizable, simplifying diagnosis. Conversely, apogeotropic forms exhibit greater variability in intensity and duration and may occasionally resemble nystagmic patterns associated with central pathologies. Therefore, it is advisable to perform positional changes multiple times, and if a transformation into a geotropic variant occurs, the diagnosis of BPPV-LSC is confirmed. However, in the absence of transformation, suspicion of a central origin is warranted.

**Apogeotropic forms of the lateral semicircular canal**

In this form of benign paroxysmal positional vertigo, the heavier material of the endolymph is located in the anterior portion of the lateral canal, either adhered to the cupula (cupulolithiasis) or freely moving within the canal (canalolithiasis). In these cases, positioning on the affected side results in an inhibitory ampullofugal current, flowing towards the healthy ear positioned upwards. When the patient lies on the healthy side, an excitatory ampullopetal current is created, always with apogeotropic nystagmus (Fig 1). In this scenario, the most intense nystagmus occurs more easily when the patient lies on the healthy side and it is more difficult to observe nystagmus evoked by simple head inclination or transition from sitting to supine. Latency is short or absent and the duration is often prolonged. The intensity is typically lower and it is very difficult to observe spontaneous nystagmus inversion. In some cases, during diagnostic maneuvers, a transformation from apogeotropic to geotropic form is observed; this indicates a displacement of otoconial debris from the anterior half to the posterior half of the lateral semicircular canal.

**
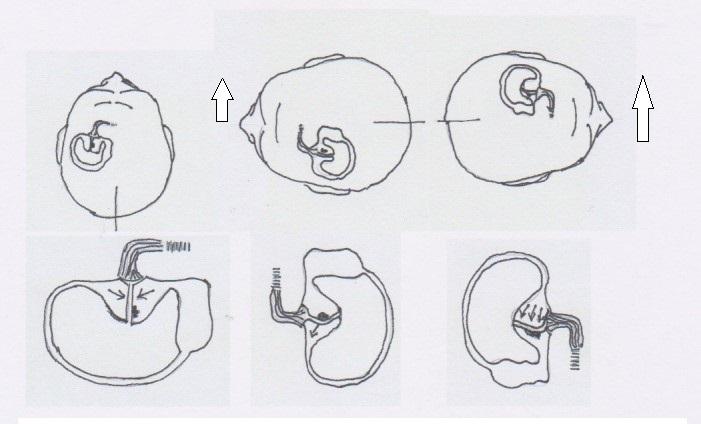
**

**Fig 1**. Paroxysmal positional peripheral vertigo of the lateral semicircular canal in apogeotropic form. Position of the head and otolithic debris within the lateral semicircular canal resulting in cupula stimulation. Arrows above indicate the direction and intensity of nystagmus

**Treatment**

**BPPV PCS therapy**

With the **Semont maneuver** [**Fig 1F**] the patient is quickly moved from a sitting position with his legs out of the table and his head rotated by approximately 45° towards the healthy side [A] to the affected side [B]. In this position, otoconial debris moves away from the ampulla of the PSC, generating an ampullifugal endolymphatic current. This induces a paroxysmal torsional nystagmus [counterclockwise for the right PSC and clockwise for the left PSC] with a vertical component upwards. The patient maintains this position until nystagmus and vertigo subside. Approximately after 60 seconds, the patient is moved to the diametrically opposite position [C], corresponding to the unaffected side, with a swift tilting movement of the trunk, ensuring the head remains in the same orientation and the transition is performed rapidly. In the lateral position corresponding to the unaffected side, a "liberatory" nystagmus, echoing the previous direction, becomes observable. This nystagmus signifies the consistent ampullifugal movement of otoconial debris towards the utricle, typically manifesting after a variable latency period. Sustaining this position for approximately 2 minutes, the patient is then slowly returned to a sitting position [D]. The examiner gradually aligns the patient's head with the body, this time taking precautions by interlocking their hands with fingers crossed behind the patient's head for a few additional seconds to prevent abrupt retropulsion.

The **Epley maneuver [Fig 1 A-E]**, also known as the "canalith repositioning maneuver," commences by placing the patient in the initiating Dix-Hallpike position, corresponding to the affected side. Subsequently, there is a gradual and continuous rotation of the patient's head towards the unaffected side until the opposite Dix-Hallpike position is achieved. At this point, the patient is instructed to lie on their side, specifically the healthy side, and further rotate the head downward until their gaze is directed towards the ground. Following a brief pause, the patient is slowly returned to a seated position.

During this movement, the examiner aims to promote the progressive migration of otoconial debris from the ampullary arm of the PSC towards the common crus and eventually into the vestibule. Importantly, this maneuver is executed without interruption, with guidance from the observed nystagmus. The nystagmus consistently maintains a torsional direction [counterclockwise for the right CSP and clockwise for the left CSP] and an upward vertical component until the otoconia exit the canal, re-entering the utricle.


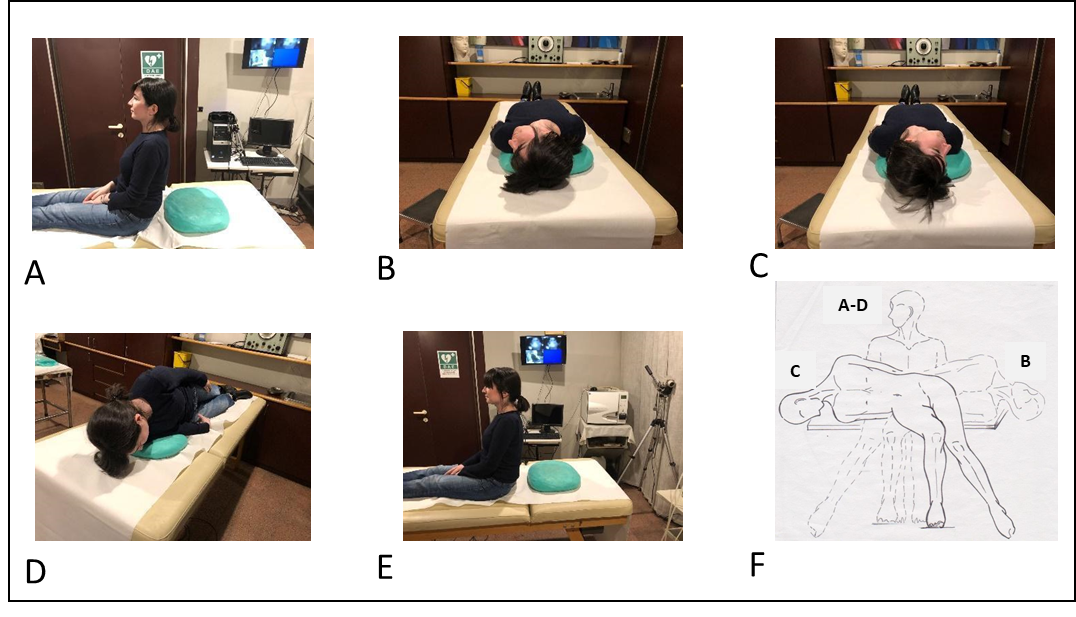


**Fig 1. Epley Maneuver for BPPV of the left PSC** [A] initiate the Epley maneuver from a seated position by turning the head 45° to the affected side [Sn] and progress to the Dix Hallpike Sn position.[B] Continue the rotation until reaching the Dix Hallpike Dx position. [C] Subsequently, turn the patient until the face is directed downwards. [D] Conclude the maneuver by bringing the patient back to a sitting position [E]. Execute each step slowly, incorporating pauses of 30" - 60". [F] **Semont Maneuver for BPPV of the left PSC**. Letters A,B,C,D inside the Fig indicate the sequential phases of the Semont maneuver.

**BPPV PCS therapy**

The **Gufoni maneuver** is done maintaining the head aligned with the body. Upon reaching the lateral position, the examiner observes for the onset of a geotropic nystagmus, indicative of effective movement of otoconial debris in an ampullifugal direction. Once reached the lateral position, the patient's head is then rotated downward by approximately 30°, with the subsequent observation of a nystagmus: also in that position nystagmus is directed in the same direction as the prior one, affirming the ongoing ampullifugal movement of debris toward the utricle. After around 30 seconds, the patient is gradually returned to a sitting position. It is advisable to repeat this sequence three to five times. Upon first positioning on the side, it is reasonable to expect dizziness and nystagmus, even if of weak intensity, since the movement is performed towards the healthy side and an inhibitory type of stimulation is generated in the canal; generally, already in the subsequent positioning, no nystagmus is observed and the patient feels less and less discomfort. During the maneuver, the affected LSC is swiftly brought into a position on the side corresponding to the healthy side, facilitating effective deceleration that propels debris towards the utricle. Subsequent downward rotation of the patient's head promotes further progression of the otoconial mass in the ampullifugal direction, positioning the non-ampullary end of the LCS in a more anti-decline orientation [Fig 2]. The Gufoni maneuver has demonstrated high efficacy and specificity in treating BPPV-LSC, proving well-tolerated even in the acute or subacute phase of vertigo due to its consistent movement toward the less symptomatic side.


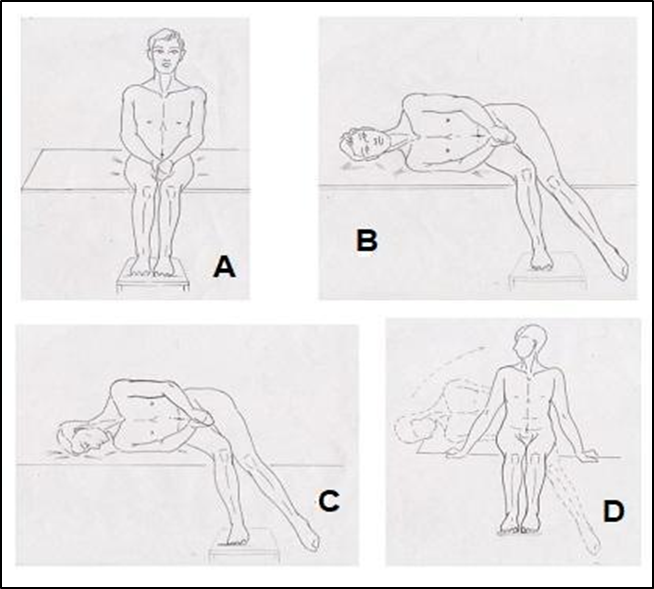


**Fig 2.** Gufoni maneuver for left-sided posterior canal BPPV
