# Supplementary material for: Consensus paper on the management of acute isolated vertigo in the emergency department
Source: Intern Emerg Med. 2024 Jul 13;19(5):1181–202. doi: 10.1007/s11739-024-03664-x (PMC11364714; doi:10.1007/s11739-024-03664-x)
Supplement: Supplementary file 4 — Diagnostic algorithm (DOCX 21 KB) [file 11739_2024_3664_MOESM4_ESM.docx]

**Diagnostic algorithm**

**The STANDING algorithm**

Firstly, the presence of spontaneous nystagmus in the supine position is evaluated using Frenzel glasses after at least 5 minutes of rest with the head in neutral position [STEP I]. If no spontaneous nystagmus is observed in the primary gaze position and there is no nystagmus upon deviating the eyes in various directions, the presence of positional nystagmus is investigated through the Pagnini-McClure maneuver and Dix-Hallpike positioning [1]. The presence of typical positional nystagmus indicates benign paroxysmal positional vertigo involving the lateral or posterior canal, respectively. Conversely, if persistent spontaneous nystagmus is present in the supine position, its direction is evaluated [STEP II]: Multidirectional, pure vertical, or torsional nystagmus is considered a sign of central vertigo. When the nystagmus is unidirectional horizontal [i.e., beating consistently to one side regardless of gaze direction and head position], the Head Impulse Test [HIT] is performed [STEP III]: a positive HIT toward the pathological side [opposite to the direction of nystagmus] suggests acute labyrinthine deficiency [peripheral], while a negative HIT [indicating good peripheral receptor function] raises suspicion of centrally originated vertigo.

Finally, in all patients, especially those without spontaneous or positional nystagmus, the ability to maintain an upright posture and walk is assessed [STEP IV]. Inability to stand or walk without assistance raises suspicion of a central pathology.

**The TI.TR.A.T.E algorithm**

Concerning the anamnestic evaluation [2,3], some authors have recently proposed an assessment utilizing timing [onset, duration, and evolution] and triggers [actions, movements, situations responsible for symptom onset] of vertigo, in addition to associated symptoms, to raise diagnostic suspicions. The targeted bedside eye examinations and any subsequent investigations [laboratory tests or imaging when a "malignant" cause is suspected] will either confirm or refute these suspicions. Associated symptoms can aid in hypothesizing medical, otovestibular or neurological problems. The **TI.TR.A.T.E.** [**T**iming, **TR**iggers, **A**nd **T**argeted **E**xamination] is a new diagnostic approach aimed at determining the probable etiology of acute vertigo, categorizing patients into four groups [2]:

- **Acute spontaneous vestibular syndrome:** sudden onset of continuous, prolonged vertigo exacerbated by head movement [but not triggered by movement], associated with vomiting, spontaneous nystagmus, postural instability [acute labyrinthine deficit generally due to vestibular neuritis in differential diagnosis with central origin, usually related to posterior circulation stroke].
- **Acute vestibular syndromes secondary** to head trauma, barotrauma, and toxicity [aminoglycosides and antiepileptics, alcohol].
- **Spontaneous episodic vertigo**: one or more episodes of transient vertigo not triggered by movement [vestibular migraine, neuro-mediated syncope, panic attack, vertebrobasilar TIA, arrhythmias, subarachnoid hemorrhage, rarely Ménière's syndrome].
- **Provoked episodic vertigo:** one or more episodes of brief positional vertigo triggered by head movement [posterior or lateral canal BPPV, orthostatic hypotension, rarely central positional vertigo indicative of lesions in the posterior cranial fossa, manifesting with atypical nystagmus patterns during positional maneuvers].

In our proposed anamnestic approach, we have omitted traumatic or toxic forms, retaining the spontaneous form, which we call Acute Vertiginous Syndrome, and Spontaneous or Triggered Episodic Vertiginous Syndrome. The subsequent pathway [clinical examination plus instrumental tests] for isolated vertigo aims to confirm diagnostic hypotheses. The **TI.TR.A.T.E** algorithm, more comprehensive than previous ones, has not been prospectively validated thus far.

**References**

1. Bhattacharyya N, Gubbels SP, Schwartz SR, Edlow JA, El-Kashlan H, Fife T, et al. Clinical Practice Guideline: Benign Paroxysmal Positional Vertigo [Update]. Otolaryngol Head Neck Surg. 2017;156[3_suppl]:S1-S47
2. Newman-Toker DE, Edlow JA. TiTrATE: A Novel, Evidence-Based Approach to Diagnosing Acute Dizziness and Vertigo. Neurol Clin. 2015 Aug;33[3]:577-99, viii. doi: 10.1016/j.ncl.2015.04.011. PMID: 26231273; PMCID: PMC4522574.
3. Newman-Toker DE, Camargo CA Jr. «Cardiogenic vertigo»--true vertigo as the presenting manifestation of primary cardiac disease. Nat Clin Pract Neurol. 2006 Mar;2[3]:167–172.
