# Supplementary material for: Consensus paper on the management of acute isolated vertigo in the emergency department
Source: Intern Emerg Med. 2024 Jul 13;19(5):1181–202. doi: 10.1007/s11739-024-03664-x (PMC11364714; doi:10.1007/s11739-024-03664-x)
Supplement: Supplementary file 5 — Diagnostic imaging (DOCX 27 KB) [file 11739_2024_3664_MOESM5_ESM.docx]

**Diagnostic imaging**

**Perfusion CT**

Perfusion imaging involves studying cerebral hemodynamics through intravenous contrast injection via an automatic injector. Under normal conditions, tissue perfusion, i.e., blood supply to tissues through capillaries, is regulated by autoregulation mechanisms. In cases of cerebral ischemia, CT perfusion [CTP] can identify the infarct core, irreversibly damaged and unsalvageable tissue, and the ischemic penumbra, the tissue surrounding the infarct core that, if rapidly reperfused, can be saved. The key perfusion parameters in ischemic cerebral pathology are cerebral blood flow [CBF] and Tmax [time to peak of the residual function curve], with threshold values of Tmax > 6 sec for the total hypoperfusion area [core + penumbra] and relative CBF [rCBF] < 30% compared to the healthy contralateral side for the infarct core [1,2].

Subsequently, perfusion maps and the CTP Tmax - CBF mismatch, fundamental for patient selection in late window clinical trials like EXTEND for intravenous rtPA treatment in the 4.5-9 hours window, DEFUSE 3 for endovascular treatment in the 6-16 hours window, and DAWN for endovascular treatment in the 6-24 hours window, are calculated [Table 1]. The calculation of perfusion maps is generally done using an automatic software.

Modern CT scanners [256-Slice CT Scanners or CT Scanners with dynamic table]cover 10 cm of brain tissue, while most available CT Scanners with 16-64 detector rows allow coverage between 2 and 4 cm. In these cases, perfusion analysis is confined to the basal ganglia to cover the territory of the middle cerebral artery, and the examination cannot include the posterior cranial fossa. The challenges of CTP in studying the posterior cranial fossa are also related to beam-hardening artifacts and the small size of infarcts, especially in forms characterized by vertigo or other isolated neurological symptoms, which may go unrecognized [3]. Achieving optimal results from perfusion imaging requires adequate training of radiologists. Consequently, these advanced techniques and the expertise required for interpreting their results are available only in select centers.

**Neuro-Imaging in acute vertigo due to suspected cerebrovascular disease**

Regarding advanced imaging recommended by national and international guidelines in the late window, two schools of thought have emerged in the last 2-3 years: 1) those considering advanced imaging highly useful/essential for selecting patients the one who considers advanced imaging very useful/indispensable for the selection of patients to undergo treatment (4-6) 2) those deeming basic imaging (CT and CTA) "sufficient," even in the late window and for patients with an unknown symptom onset time (7-11).

When selecting neuro-imaging tests, it is crucial to differentiate between first-level centers (spokes), obligated to provide at least basic imaging in accordance with local protocols, and second-level centers (hubs), equipped with all imaging techniques, both basic and advanced. These centers should possess the requisite expertise to discern the therapeutic window for various acute treatments, as outlined in the international (AHA/ASA 2018) (12) and national guidelines (ISO SPREAD 2020)(13). This is particularly pertinent following the recent dissemination of findings from clinical trials such as DAWN (14), DEFUSE 3 (15), Wake up (16), and EXTEND (17). For patients with an unknown onset time of symptoms, the advent of new neuroimaging techniques has introduced the possibility of intravenous thrombolysis and endovascular treatment (12).

Diagnostic methods include CT, including preferably multiphase Angio-CT (mCTA), CTP, and MRI with Angio-MRI, DWI, and PWI techniques. The choice of method and technique depends on the time window and the local organization of individual centers. [Table 2]

Patient selection through basic imaging (NCCT and CTA preferably multiphaseCTA) in the late window offers the advantage of reaching a higher number of ischemic stroke patients, even in centers without advanced imaging techniques.

The recent results of the MR CLEAN-LATE clinical trial (11) have extended the possibility of selecting patients with suspected stroke within the 6-24-hour window solely through CTA with collateral circulation assessment.

**Table 1.** Treatment Selection Criteria for Ischemic Stroke in Key Clinical Studies

| **TRIAL** | **Method/Technique** | **Selection criteria** | **Selection criteria** |
| --- | --- | --- | --- |
|  |  |  |  |
| EXTEND 2019 [17] | CTP/MRI | - core volume < 70 ml - penumbra volume > 10 ml | - mismatch ratio > 1.2 |
| DAWN 2018 [14] | CTP  MRI DWI | - core volume <21 ml if >80 years; NIHSS ≥ 20 - core volume < 31 ml if < 80 years; NIHSS ≥10 - core volume < 31-51 ml if < 80 years; NIHSS ≥20 |  |
| DEFUSE 3 2018 [15] | CTP/MRI | - core volume < 70 ml - penumbra volume > 15 ml | - mismatch ratio >1.8 |
| WAKE-UP 2018 [16] | MRI DWI/FLAIR | - DWI+/FLAIR - - DWI+/FLAIR + | - presence of mismatches - absence of mismatches |

**Radiological imaging indications in patients with suspected acute cerebrovascular disease [18] [Table 2]**

1) In case of suspected acute stroke within the 0-4.5-hour window (the only one recommended by alteplase's technical data), performing a baseline non-contrast CT scan is advised to exclude intra- and extraparenchymal hemorrhages and stroke mimics such as expansive processes (Strong Recommendation).

2) In cases of suspected acute stroke eligible for intravenous rtPA treatment within the extended 4.5-9-hour window based on the EXTEND trial results (2019), selecting patients with CT, CTA, and CTP scans with a mismatch ratio of 1.2 is recommended (Strong Recommendation).

3) Within the 0-6-hour window for patients eligible for endovascular treatment, selecting patients with baseline CT and CTA scans to identify vascular occlusion and evaluate collateral circulation is advised (Strong Recommendation).

4-5) In the late window (6 up to 24 hours) for patients eligible for endovascular treatment, patient selection via CTP according to DAWN and DEFUSE 3 trial criteria is recommended (Strong Recommendation)

6) In patients with suspected stroke and unknown symptom onset time or upon awakening, it is strongly recommended to select patients for rtPA treatment or according to the EXTEND trial criteria via CTP (core <70 ml, penumbra >15 ml, target mismatch 1.2) or according to the Wakeup trial criteria via MRI with DWI and FLAIR sequences to date the stroke within the 4.5-hour window (positive DWI and negative FLAIR = presence of mismatch, likely less than 4.5 hours, while positive DWI and positive FLAIR = absence of mismatch, likely more than 4.5 hours). The choice of method depends on local organization.

**Table 2.** Therapeutic Windows in Ischemic Stroke and Neuro-Imaging

| 1. 0-4.5 hours for rtPA thrombolysis: CT and CTA 2. 4.5-9 hours for rtPA thrombolysis: CT, CTA, CTP 3. 0-6 hours for endovascular treatment: CT and CTA 4. 6-16 hours for endovascular treatment: CT, CTA, CTP 5. 6-24 hours for endovascular treatment: CT, CTA, CTP 6. Unknown time of onset: CT, CTA, CTP, MRI (DWI/FLAIR) |
| --- |

**Neuro-imaging in acute vertigo and GRACE-3 Guidelines [19]**

**Recommendation 5**: In adult patients with acute vestibular syndrome (AVS) with or without nystagmus, we do not recommend the routine use of non-contrast CT scan or CT angiography to distinguish between central (stroke) and peripheral vertigo (Strong Recommendation Against).

**Recommendation 6**: In adult patients with acute vestibular syndrome (AVS) with or without nystagmus, we do not recommend the routine use of MRI or MR angiography as a first-line test (prior to clinical and objective examination) to distinguish between central (stroke) and peripheral vertigo (Strong Recommendation Against).

**Recommendation 7**: In adult patients with acute vestibular syndrome with suggestive results for central origin on the HINTS test, we recommend performing MRI with DWI sequence and MR angiography to add diagnostic elements to the differential diagnosis between central and peripheral vertigo (Strong Recommendation in Favor).

**Recommendation 9**: In adult patients with spontaneous episodic vestibular syndrome (s-EVS), routine use of CT scan to differentiate between central vertigo (TIA), benign central vertigo (vestibular migraine), and peripheral vertigo is not recommended (Strong Recommendation Against).

**Recommendation 10**: In adult patients with s-EVS and strong suspicion of TIA, it is suggested to perform CT angiography or MR angiography to exclude a vascular pathology of the posterior circulation (Weak Recommendation in Favor).

**Recommendation 12**: In adult patients with provoked episodic vestibular syndrome (triggered-EVS), routine use of CT scan and CT angiography is not recommended (Strong Recommendation Against).

**Recommendation 13**: In adult patients with provoked episodic vestibular syndrome (triggered-EVS) with benign paroxysmal positional vertigo diagnosed by the Dix-Hallpike test with characteristic nystagmus, routine use of MRI and MR angiography is not recommended (Weak Recommendation Against).

**References**

1. Olivot JM et al. Optimal Tmax Threshold for Predicting Oenumbral Tissue in Acute Stroke. Stroke 2009; 40[2]: 469-75
2. Campbell BCV et al. Cerebral Blood Flow Is the Optimal CT Perfusion Parameter for Assessing Infarct Core. Stroke 2011; 42: 3435-40
3. Kargiotis O et al. Computed Tomography Perfusion Imaging in Acute Ischemic Stroke: Accurate Interpretation Matters. Stroke 2023; 54: 104-108[Powers](https://www.ahajournals.org/doi/10.1161/STR.0000000000000158), Rabinstein, Ackerson, [Adeoye](https://www.ahajournals.org/doi/10.1161/STR.0000000000000158) and on behalf of the American Heart Association Stroke Council, Guidelines for the Early Management of Patients With Acute Ischemic Stroke: A Guideline for Healthcare Professionals From the American Heart Association/American Stroke Association, 2018 Stroke. 2018;49:e46–e99
4. Tsivgoulis G et al. Advanced Neuroimaging in Stroke Patient Selection for Mechanical Thrombectomy. Stroke 2018; 49: 3067-3070
5. Albers GW et al. Assessment of Optimal Patient Selection for Endovascular Thrombectomy Beyond 6 Hours After Symptom Onset. JAMA Neurol 2021; 78: 1064-1071
6. Tan Z et al. Comparison of Computed Tomography Perfusion and Multiphase Computed Tomography Angiogram in Predicting Clinical Outcomes in Endovascular Thrombectomy. Stroke 2022; 53: 2926-2934
7. Almekhlafi MA et al. Imaging Triage of Patients with Late-Window [6-24 Hours] Acute Ischemic Stroke : A Comparative Study Using Multiphase CT Angiography versus CT Perfusion. Am J Neuroradiol 2020; 41: 129-133
8. Nogueira RG et al. Stroke Imaging Selection Modality and Endovascular Therapy Outcomes in the Early and Extended Time Windows. Stroke 2021; 52: 491-497
9. Bouslama M et al. Novel selection paradigms for endovascular stroke treatment in the extended time window. J Neurol Neurosurgery Psychiatry 2021; 92: 1152-1157
10. Nguyen TN et al. Noncontrast Computed Tomography vs Computed Tomography Perfusion or Magnetic resonance Imaging Selection in Late Presentation of Stroke With Large-Vessel Occlusion. JAMA Neurol 2022; 79:22-31
11. Olthuis SGH et al. Endovascular treatment versus no endovascular treatment after 6-24 h in patients with ischaemic stroke and collateral flow on CT angiography [MR CLEAN-LATE] in the Netherlands: a multicentre open-label, blinded-endpoint, randomized, controlled, phase 3 trial. Lancet 2023; 401: 1371-1380
12. [Powers](https://www.ahajournals.org/doi/10.1161/STR.0000000000000158), Rabinstein, Ackerson, [Adeoye](https://www.ahajournals.org/doi/10.1161/STR.0000000000000158) and on behalf of the American Heart Association Stroke Council, Guidelines for the Early Management of Patients With Acute Ischemic Stroke: A Guideline for Healthcare Professionals From the American Heart Association/American Stroke Association, 2018 Stroke. 2018;49:e46–e99
13. ISO-SPREAD Guidelines 2020
14. Gregory W. Albers, M.D., Michael P. Marks, Thrombectomy for Stroke at 6 to 16 Hours with Selection by Perfusion Imaging M.D. N Engl J Med 2018; 378:708-718
15. Götz Thomalla, M.D., Claus Z. Simonsen, M.D. MRI-Guided Thrombolysis for Stroke with Unknown Time of Onset. N Engl J Med 2018; 379:611-622
16. Sankalia D, Kothari S, Phalgune DS. Diagnosing stroke in Acute Vertigo: Sensitivity and Specificity of HINTS Battery in Indian Population. Neurol India 2021; 69: 97-101
17. Raul G. Nogueira, M.D., Ashutosh P. Jadhav,Thrombectomy 6 to 24 Hours after Stroke with a Mismatch between Deficit and Infarct. M.D. N Engl J Med 2018; 378:11-21
18. Stroke Prevention and Educational Avareness Diffusion guidelines, 2020.
19. [Jonathan A Edlow](https://pubmed.ncbi.nlm.nih.gov/?sort=pubdate&term=Edlow+JA&cauthor_id=37166022), Christopher Carpenter, [Murtaza Akhter](https://pubmed.ncbi.nlm.nih.gov/?sort=pubdate&term=Akhter+M&cauthor_id=37166022) et al. Guidelines for reasonable and appropriate care in the emergency department 3 [GRACE-3]: Acute dizziness and vertigo in the emergency department, 2023.
