# Supplementary material for: Consensus paper on the management of acute isolated vertigo in the emergency department
Source: Intern Emerg Med. 2024 Jul 13;19(5):1181–202. doi: 10.1007/s11739-024-03664-x (PMC11364714; doi:10.1007/s11739-024-03664-x)
Supplement: Supplementary file 6 — Early secondary prophylaxis (DOCX 17 KB) [file 11739_2024_3664_MOESM6_ESM.docx]

**Early secondary prophylaxis**

Individuals experiencing ischemic stroke or transient ischemic attack [TIA] face a heightened likelihood of early recurrence. Findings from randomized trials propose that initiating early secondary prophylaxis with antiplatelet agents [specifically aspirin at a dosage of 160-300 mg per day] is advisable for all patients with ischemic stroke or TIA [1]. In cases where patients are undergoing revascularization therapies, the commencement of antiplatelet therapy should occur 24 hours post-procedure, following a confirmatory brain CT scan to rule out hemorrhagic transformations.

Additional randomized studies propose specific interventions for patients at high risk of early recurrence following transient ischemic attack [TIA]. Notably, those with a TIA at high risk [ABCD2 score >3] or presenting with a mild stroke [NIHSS < 4] of non-cardioembolic origin within 24 hours may benefit from dual antiplatelet therapy, involving clopidogrel [300-600 mg loading dose on the first day, followed by 75 mg] and aspirin [100-300 mg daily] [2]. The optimal duration for this dual antiplatelet regimen, balancing efficacy and safety, appears to be 21 days [3].

For individuals with TIA or minor stroke from cardioembolic causes early initiation of oral anticoagulants might be considered [4]. It is crucial to recognize that patients with posterior circulation stroke often exhibit low NIHSS scores, designed for anterior circulation strokes. Therefore, careful consideration is warranted, especially when symptoms such as isolated vertigo and ataxia due to a cerebellar lesion may yield a low NIHSS, classifying the patient as experiencing a minor stroke and possibly eligible for dual antiplatelet therapy. However, it's essential to assess each case individually, as, for example, a patient within the thrombolysis time window exhibiting significant disability, such as the inability to stand and walk, should be considered for thrombolysis, despite a seemingly low NIHSS.

**References**

1. Chen ZM, Sandercock P, Pan HC, Counsell C, Collins R, Liu LS, Xie JX, Warlow C, Peto R. Indications for early aspirin use in acute ischemic stroke : A combined analysis of 40 000 randomized patients from the chinese acute stroke trial and the international stroke trial. On behalf of the CAST and IST collaborative groups. Stroke. 2000 Jun;31[6]:1240-9
2. Trifan G, Gorelick PB, Testai FD. Efficacy and Safety of Using Dual Versus Monotherapy Antiplatelet Agents in Secondary Stroke Prevention: Systematic Review and Meta-Analysis of Randomized Controlled Clinical Trials. Circulation. 2021 Jun 22;143[25]:2441-2453
3. [Qiukui Hao](https://pubmed.ncbi.nlm.nih.gov/?sort=pubdate&term=Hao+Q&cauthor_id=30563866), [Malavika Tampi](https://pubmed.ncbi.nlm.nih.gov/?sort=pubdate&term=Tampi+M&cauthor_id=30563866), [Martin O'Donnell](https://pubmed.ncbi.nlm.nih.gov/?sort=pubdate&term=O'Donnell+M&cauthor_id=30563866), [Farid Foroutan](https://pubmed.ncbi.nlm.nih.gov/?sort=pubdate&term=Foroutan+F&cauthor_id=30563866), [Reed Ac Siemieniuk](https://pubmed.ncbi.nlm.nih.gov/?sort=pubdate&term=Siemieniuk+RA&cauthor_id=30563866), [Gordon Guyatt](https://pubmed.ncbi.nlm.nih.gov/?sort=pubdate&term=Guyatt+G&cauthor_id=30563866). Clopidogrel plus aspirin versus aspirin alone for acute minor ischaemic stroke or high risk transient ischaemic attack: systematic review and meta-analysis.BMJ.. 2018 Dec 18:363:k5108.
4. Seiffge DJ, Werring DJ, Paciaroni M, Dawson J, Warach S, Milling TJ, Engelter ST, Fischer U, Norrving B. Timing of anticoagulation after recent ischaemic stroke in patients with atrial fibrillation. Lancet Neurol. 2019 Jan;18[1]:117-126.
