# Supplementary material for: Consensus paper on the management of acute isolated vertigo in the emergency department
Source: Intern Emerg Med. 2024 Jul 13;19(5):1181–202. doi: 10.1007/s11739-024-03664-x (PMC11364714; doi:10.1007/s11739-024-03664-x)
Supplement: Supplementary file 7 — Focused physical examination (DOCX 21 KB) [file 11739_2024_3664_MOESM7_ESM.docx]

**Focused physical examination**

**General concepts**

In the first phase, the coexisting alteration of vital parameters and the presence of other general [such as pale skin, sweating] or cardiovascular symptoms [like heart palpitations, chest pain, dyspnea] guide the diagnostic process. These findings need to be confirmed by a targeted objective examination.

In an emergency, the correct methodology to guide the diagnosis involves focusing on the prevalent objective symptom or sign. For instance, pale skin may indicate anemia, while sweating and mental confusion may accompany a hypoglycemic crisis. If vertigo is the prevalent symptom, it is important to note that not all vertigo disorders originate from the vestibular system, whether peripheral or central. Recent studies indicate that most patients presenting with vertigo or unsteadiness do not have vestibular disease [1].

Pseudo-dizziness, a term denoting non-vestibular sensations of imbalance, encompasses conditions wherein approximately one-third are linked to potentially dangerous pathological conditions such as severe hemorrhage, profound hypoglycemia, and brady or hemodynamic tachyarrhythmia [see Table 1]. Pseudovertigo includes psychogenic disorders [anxiety, depression, panic attacks, conversion disorder] often marked by hyperventilation. A comparable sensation of imbalance is evident during alcoholic intoxication, be it occasional or habitual, as well as the subsequent day following excessive alcohol exposure, albeit in a more nuanced manner. In the context of severe anemia, asthenia upon standing is more appropriately considered an epiphenomenon of hemoglobin deficiency rather than a lack of balance. The finding of orthostatic hypotension associated with a reported pre-syncopal or even dizziness-like symptoms supports the anamnestic diagnosis of transient episodes exclusively occurring in an orthostatic position. On the orher side, Persistent dizziness coupled with headache while standing may indicate cerebrospinal fluid [CSF] hypotension, warranting thorough investigation. The anamnestic finding of heart palpitations or pressure on the anterior region of the neck precipitating the dizziness disorder merit an electrocardiographic examination and the exploration of extreme bradycardia induced by carotid sinus massage. Recurrent partial epilepsy disorders, lacking true tonic-clonic seizures and morsus, can mimic sensations of instability or reported rotation unrelated to movement. These manifestations are accompanied by additional neurological signs, including altered consciousness and sensory and/or motor disturbances.

The concurrent occurrence of a disorder among individuals residing together raises concerns about carbon monoxide [CO] intoxication, capable of inducing dizziness independent of CO-hemoglobin levels. Metabolic disorders such as hyponatremia or hypercapnia can be ruled out through laboratory findings, particularly in individuals undergoing chronic diuretic therapy or those with obstructive pulmonary disease and/or sleep apnea syndrome. Hypomagnesemia may elude detection if not actively suspected, given its absence in routine urgent blood tests. Nevertheless, in the presence of a dizziness disorder accompanied by central-type nystagmus, thorough investigation for hypomagnesemia is warranted.

**Neurological examination**

The presence of at least one of these signs should be considered indicative of a potentially central origin of vertigo. Emphasis should be placed on the identification of cerebellar signs, bulbar cranial nerve signs, and Bernard-Horner syndrome, as they are closely associated with vestibular pathways and frequently coincide with vertigo [2]. Additionally, meticulous attention should be given to the detection of sensory disorders. Central vertigo may arise from a lesion of the bulbar vestibular nuclei, situated in proximity to the descending tracts of superficial sensation. Due to the somatotopic distribution of sensory pathways at this level, hypoesthesia may manifest, possibly affecting only the lower limb. This deficit could be overlooked if sensory evaluations are limited to the upper limbs.

***Cerebellar tests***

The cerebellum comprises three main regions: a central area called the cerebellar vermis [spino-cerebellar], on the sides of which there are two cerebellar hemispheres [cerebro-cerebellum] and below the flocculo-nodular lobe [vestibulo-cerebellum]. Between the vermis and the cerebellar hemispheres, there is a thin part of cerebellar tissue called the intermediate zone. This anatomical subdivision enables the identification of distinct deficits based on the affected area. even if all the parts are connected to each other with the possibility of overlapping clinical pictures

***Muscle tone and posture***

Damage to the cerebellar vermis and the intermediate area [spino-cerebellum] primarily manifests as impaired control of muscle tone and posture. From a semiological perspective, the effects of spinocerebellar damage can be identified through:

- muscle tone: Muscular hypotonia is evident in muscles ipsilateral to the lesion due to the absence of cerebellar tonic influence on motor neurons. The reduction in muscle tone can be assessed using the "Stewart-Holmes rebound test."
- standing and walking: When prompted to maintain an upright position and tilt the trunk backward, the patient exhibits a tendency to fall [asynergy of standing]. Additionally, during walking, the patient fails to lean the trunk forward, leading to a backward tendency and an increased risk of falling.

***Motor coordination***

Injury to the cerebellar hemispheres [cerebro-cerebellum] results in challenges initiating and coordinating movements with the limbs. This includes manifestations such as dysmetria [hypermetria, hypometria] and dysdiadochokinesia, characterized by a diminished ability to execute rapid alternating movements.

Symptoms arising from cerebellar hemisphere damage are ipsilateral to the lesion. For instance, damage to the left cerebellar hemisphere induces dysmetria in the left limbs.

**G*ait control***

Damage to the vestibule-cerebellum [flocculus-nodular lobe] causes disturbances in balance and eye movements. Clinically in cerebellar syndrome we distinguish:

- Cerebellar ataxia: is defined as both static and dynamic, impacting both standing and walking [3,4]. The disturbances in balance and walking are not corrected by sight [as opposed to patients with sensory ataxia], with a negative Romberg test [cerebellar patients already exhibit oscillations or lateral pulsion with open eyes, which are not significantly influenced by closing the eyes, unlike patients with proprioceptive pathway lesions]. When assessing patients with isolated vertigo, it is crucial to evaluate their ability to maintain standing and walking. The greater the difficulty in sustaining these activities, the higher the likelihood that the vertigo originates centrally [refer to Table 2].

***Other signs of cerebellar injury***

In addition to the aforementioned signs, patients exhibiting cerebellar syndrome may manifest the following:

- Dysarthria: speech alteration attributed to an incoordination of involved muscles, distinct from the conventional dysarthria resulting from anterior circulation dysfunction.
- Intention tremor: cerebellar tremor manifests during voluntary movements towards a target. The amplitude is notably high, with a low frequency throughout the complete movement. The tremor intensifies as the target approaches, as observed in the Finger-to-Nose test.
- Alterations of ocular motility, including disruptions in smooth pursuit and saccadic systems, are detailed in the oculomotor study chapter.

**References**

1. David E Newman-Toker, [Yu-Hsiang Hsieh](https://pubmed.ncbi.nlm.nih.gov/?sort=pubdate&term=Hsieh+YH&cauthor_id=18613993), [Carlos A Camargo Jr](https://pubmed.ncbi.nlm.nih.gov/?sort=pubdate&term=Camargo+CA+Jr&cauthor_id=18613993), [Andrea J Pelletier](https://pubmed.ncbi.nlm.nih.gov/?sort=pubdate&term=Pelletier+AJ&cauthor_id=18613993), [Gregary T Butchy](https://pubmed.ncbi.nlm.nih.gov/?sort=pubdate&term=Butchy+GT&cauthor_id=18613993), [Jonathan A Edlow](https://pubmed.ncbi.nlm.nih.gov/?sort=pubdate&term=Edlow+JA&cauthor_id=18613993). Spectrum of dizziness visits to US emergency departments: cross-sectional analysis from a nationally representative sample Mayo Clin Proc. 2008 Jul;83[7]:765-75
2. Kerber KA, Meurer WJ, West BT, Fendrick AM. Dizziness presentations in U.S. emergency departments, 1995-2004. Acad Emerg Med. 2008;15[8]:744-50.
3. Diener HC, Dichgans J. Pathophysiology of cerebellar ataxia. Movement Disorders 1992;7:95-109.
4. Holmes G. The symptoms of acute cerebellar injuries due to gunshot injuries. Brain 1917;40:462-535
