# Supplementary material for: Consensus paper on the management of acute isolated vertigo in the emergency department
Source: Intern Emerg Med. 2024 Jul 13;19(5):1181–202. doi: 10.1007/s11739-024-03664-x (PMC11364714; doi:10.1007/s11739-024-03664-x)
Supplement: Supplementary file 8 — Medical History (DOCX 24 KB) [file 11739_2024_3664_MOESM8_ESM.docx]

**Medical History**

**Acute vertiginous syndrome**

**Pathophysiology**

The most frequent peripheral cause of the acute vertiginous syndrome is DVA, generally attributed to vestibular neuritis [1-3]. It is important to point out that the term "labyrinthitis", with which DVA is often improperly defined, must be used for those rare inflammations/infections affecting the middle ear and spreading to the inner ear, with the involvement of the vestibule and the cochlea. There is also a "labyrinthitis" secondary to meningitis and in this case it can be masked by the predominant meningeal neurological symptoms.

The viral origin, or more precisely, the hypothesis implying a reactivation of a latent neurotropic virus in the vestibular ganglia is considered the most common. This hypothesis seems to be supported by the frequent finding in the medical history of recent inflammation of the upper respiratory tract. Nevertheless, an inflammatory process affecting the vestibular nerve is considered highly probable if the onset of vertiginous crisis is preceded or accompanied by other viral manifestations generally attributable to neurotropic viruses such as Herpes Simplex type 1 or Herpes Zoster (i.e., the appearance of vesicles on the involved side of the face, particularly on the external auditory canal or the auricular pavilion on the same side) or by more generic symptoms attributable to influenza or parainfluenza viruses, mumps, rubella, and even SARS-CoV-2 itself. If a definite diagnosis is possible only through the detection of viral DNA in the context of the vestibular nerve itself [4], the presence of enhancement at the level of the vestibular nerve ganglion in magnetic resonance with gadolinium in the acute phase of a vestibular neuritis attack represents a strongly suggestive element of the diagnosis [5]. The superior vestibular nerve, within which the afferents from the aforementioned receptors run, is more commonly involved by NV since the superior branch of the vestibular nerve, inside which the afferents from the aforementioned receptors run, presents greater vulnerability due to intrinsic anatomical factors [6,7].

On the other hand, the pathogenetic mechanism of vascular type appears to be determined by occlusion of the anterior (or superior) vestibular artery. It is the terminal branch of the labyrinthine artery (or internal auditory artery) that primarily supplies the same receptors innervated by the superior branch of the vestibular nerve [8]. This pathogenetic mechanism cannot be excluded a priori, considering that the inner ear is supplied by terminal arterial branches and central structures involved in maintaining posture and balance are extremely sensitive to decreases in blood flow. This hypothesis seems to be supported by the increased risk of stroke in patients hospitalized for vertigo. (8,9) Therefore a micro-ischemic mechanism, even if peripheral, should be suspected in elderly patients and those with evident risk factors such as hypertension, obesity, diabetes, heart disease, and previous cerebrovascular ischemic events when sudden onset of vestibular dysfunction occurs, as in other vascular origins. In cases of micro-ischemic vestibular dysfunction, it is not uncommon to observe subsequent detachment of otolithic debris from the utricular macula, resulting in canalolithiasis of the semicircular canal still functionally active due to being spared by the pathogenic insult. [10,11].

**Classification of Acute Vestibular Deficit**

**Classification according to the Barany Society [1]**

**1) Acute unilateral vestibulopathy**

A) Acute or subacute onset of sustained rotatory or non-rotatory vertigo of moderate to severe intensity with symptoms lasting longer than 24 hours.

B) Spontaneous nystagmus with a direction congruent with the input of the involved semicircular canals, generally horizontal-torsional, with a fixed direction and increased by visual fixation removal.

C) Unequivocal evidence of reduced vestibulo-ocular reflex (VOR) function on the side opposite to the direction of the fast phase of spontaneous nystagmus.

D) No evidence of acute neurological, otological, or audiological symptoms.

E) Absence of acute signs compatible with central nervous system alteration, i.e., absence of central oculomotor or central vestibular signs, particularly absence of pronounced skew deviation, gaze-evoked nystagmus, or acute audiological or otological signs.

F) Symptoms not better explained by another pathology.

**2) Acute evolving unilateral vestibulopathy**

A) Acute or subacute onset of sustained rotatory or non-rotatory vertigo, with symptoms lasting for more than 3 hours but for a period of time shorter than 24 hours at the time the patient is examined.

B) Symptoms not better explained by another pathology.

**3) Probable acute unilateral vestibulopathy**

Essentially identical to the first category, except that the unilateral deficit of the VOR is not clearly observed or documented.

**4) History of acute unilateral vestibulopathy**

A) History of acute or subacute onset of vertigo lasting at least 24 hours and slowly decreasing in intensity.

B) No history of acute neurological symptoms or simultaneous symptoms of the central nervous system.

C) Unequivocal evidence of unilaterally reduced VOR function.

D) No history of simultaneous acute signs indicative of central nervous system alteration, i.e., no central oculomotor signs or central vestibular signs, and no acute audiological or otological signs.

E) Symptoms not better explained by another pathology

**Episodic vertiginous syndrome**

**Spontaneous episodic vertiginous syndrome [s-EVrS]**

In the case of VM, there are no pathognomonic signs. Nystagmus, when present, can be peripheral, central, or mixed. Therefore, the diagnosis relies on the medical history and the exclusion of other diseases.

Patients with MD commonly exhibit recurrent episodic vertigo, often accompanied by tinnitus and a sensation of fullness in the ear. Hearing loss is also a frequent symptom, and in the initial stages, it may be reversible. However, at the onset of the disease, only one in four patients presents with all these symptoms.

Panic attacks can be associated with episodic dizziness and the fear of dying. These attacks typically have a rapid onset and reach their maximum intensity within about 10 minutes.

Certain forms of temporal lobe epilepsy can manifest with transient dizzy episodes, often referred to as vertiginous auras. These episodes typically last only a few seconds or minutes and are commonly associated with an alteration of mental status.

Vasovagal syncope typically presents with prodromes, and among these, vertigo is the most common symptom and can manifest in various forms [41]. Notably, pre-syncope episodes, characterized by the absence of loss of consciousness, are more frequent than true syncopal episodes. Consequently, many patients may report isolated episodes of vertigo. The diagnosis relies on clinical history and the exclusion of other potentially dangerous pathologies.

Vertigo is the most frequent prodromal symptom of vertebrobasilar TIA, particularly when associated with other neurological symptoms. It is more common in the period ranging from a few days to a few weeks before the ischemic stroke of the posterior circulation. Additionally, vertigo is the most prevalent presenting symptom of vertebral artery dissection. In patients with s-EVrS, the presence of at least three vascular risk factors or an ABCD_2_ score of at least 4 is predictive of TIA. Moreover, cardiac arrhythmias should be considered in all patients with s-EVrS, especially if syncope or dizziness occurs during exertion.

**Triggered episodic vertiginous syndrome [t-EVrS]**

Distinguishing triggers [head or body movements that induce symptoms not present in basal conditions] from aggravating factors [head or body movements that worsen pre-existing symptoms] is crucial. It's important to note that the movement of the head can accentuate all types of vertigo, whether of central or peripheral origin, acute or chronic. If vertigo worsening with head movements is indicative of a peripheral type is a common misconception.

Special attention should be given to vertigo induced by changes in position, as orthostatic dizziness may not always be linked to the presence of orthostatic hypotension. This condition could be attributed to BPPV but may also indicate a hemodynamic TIA resulting from hypoperfusion distal to an intracranial vascular stenosis. Patients experiencing reproducible and sustained orthostatic vertigo without documentable hypotension or BPPV should undergo a neurological evaluation.

**References**

1. Strupp M, Bisdorff A, Furman J, et al. Acute unilateral vestibulopathy/vestibular neuritis: Diagnostic criteria. *J Vestib Res*. 2022;32(5):389-406.
2. Baloh RW, Ishyama A, Wackym PA, Honrubia V. Vestibular neuritis: clinical-pathologic correlation. *Otolaryngol Head Neck Surg*. 1996;114(4):586-592.
3. Jeong SH, Kim HJ, Kim JS. Vestibular neuritis. *Semin Neurol*. 2013;33(3):185-194
4. Arbusow V, Derfuss T, Held K. Latency of herpes simplex virus type-1 in human geniculate and vestibular ganglia is associated with infiltration of CD8+ T cells. *J Med Virol.* 2010; 82: 1917–1920
5. Venkatasamy A, Huynh TT, Wohlhuter N, et al. Superior vestibular neuritis: improved detection using FLAIR sequence with delayed enhancement (1 h). *Eur Arch Otorhinolaryngol.* 2019;276(12):3309-3316.
6. Fetter M, Dichgans J. Three-dimensional human VOR in acute vestibular lesions. *Ann N Y Acad Sci* 1996;781:619–621.
7. Gianoli G, Goebel J, Mowry S, Poomipannit P. Anatomic differences in the lateral vestibular nerve channels and their implications in vestibular neuritis. *Otol Neurotol* 2005;26:489-94
8. Kim JS, Lee H. Inner ear dysfunction due to vertebrobasilar ischemic stroke. *Semin Neurol* 2009;29:534-40
9. Lee CC, Ho HC, Su YC, et al. Increased risk of vascular events in emergency room patients discharged home with diagnosis of dizziness or vertigo: a 3-year follow-up study. *Plos One* 2012;7:e35923.
10. Lindsay JR, Hemenway WG. Postural vertigo due to unilateral sudden partial loss of vestibular function. *Arch Otolaryngol* 1956;65:692–706
11. Casani AP, Cerchiai N, Navari E. Paroxysmal positional vertigo despite complete vestibular impairment: the role of instrumental assessment. *Acta Otorhinolaryngol Ital*. 2018;38(6):563-568
