# Supplementary material for: Consensus paper on the management of acute isolated vertigo in the emergency department
Source: Intern Emerg Med. 2024 Jul 13;19(5):1181–202. doi: 10.1007/s11739-024-03664-x (PMC11364714; doi:10.1007/s11739-024-03664-x)
Supplement: Supplementary file 9 — Methods (DOCX 16 KB) [file 11739_2024_3664_MOESM9_ESM.docx]

**Methods**

The authors of the document, coming from various Italian hospitals and universities, met by invitation of the Study Group of the Italian Society of Vestibology (VIS) and the Study & Research Center of the Italian Society of Emergency Medicine (SIMEU)..

The Working Group has identified the following general objectives to be developed:

1. Define the main anamnestic and clinical criteria that are useful for the differential diagnosis of patients who access the ED for acute vertigo.
2. Clarify the role of diagnostic exams, in particular diagnostic imaging, describing their indications and limitations.
3. Provide a useful scheme for defining the diagnostic, therapeutic and care management of these patients.

After having identified the main questions, a document was developed during various meetings from March to June 2023 referring point by point to the available scientific evidence. During the meetings, each chapter was summarized in essential points reported in special text boxes (key messages). These were discussed and submitted for approval in detail by the Working Group. The points reported in the key messages were approved unanimously or put to the vote and approved with a degree of consensus greater than 75% of members.

At the end of the drafting, the manuscript was subjected to further review by experts in the two disciplines external to the Working Group. After this peer review phase of the text, the comments were discussed by the Working Group and included in the document that was sent for approval to the national governing councils of the scientific societies VIS and SIMEU.

 The consensus document was presented by the Working Group to a large audience of professionals both in the discipline of emergency medicine and audiology on the occasion of national conferences organized by the respective scientific societies.
