# Supplementary material for: Consensus paper on the management of acute isolated vertigo in the emergency department
Source: Intern Emerg Med. 2024 Jul 13;19(5):1181–202. doi: 10.1007/s11739-024-03664-x (PMC11364714; doi:10.1007/s11739-024-03664-x)
Supplement: Supplementary file 10 — Nystagmus Patterns of Central Origin (DOCX 17 KB) [file 11739_2024_3664_MOESM10_ESM.docx]

**Nystagmus Patterns of Central Origin**

Firstly, we will describe *nystagmus induced by ocular maneuvers.* Shifting a subject's eyes to extreme lateral positions often reveals a nystagmus beating in the direction of gaze deviation. This nystagmus is para-physiological, tends to fatigue, and is more challenging to observe under fixation; in this case, it is termed ‘extreme eye gaze nystagmus’ or ‘end-point nystagmus’. In contrast, the presence of nystagmus appearing with a gaze deviation of 30° [no more than 45°] signifies central disorder, affecting the neural integrator of gaze, primarily located at the medial vestibular nucleus and the prepositus hypoglossal nucleus [1]. This is termed ‘eccentric gaze nystagmus’ or ‘*gaze-evoked nystagmus'*, which can be unilateral or bilateral, does not fatigue and is better observed under fixation. Furthermore, after asking the patient to gaze laterally for at least 20 seconds and then return their eyes to the center, a normal subject would not exhibit nystagmus. However, in cases of archi-cerebellar involvement, a ‘rebound nystagmus’ may occur, where nystagmus appears in the opposite direction upon returning to the center [2]. It can be unilateral or bilateral. The origin of these nystagmus is almost always central, necessitating further investigation.

Observing *spontaneous vertical nystagmus*, either upward or downward, is another sign of likely central origin, especially if observed under fixation and without head movements. The fast phase tends to indicate the location of the lesion; thus, a downward nystagmus suggests a lesion in the bulbo-pontine region [e.g., Arnold-Chiari syndrome], while an upward nystagmus indicates ponto-mesencephalic involvement.

There is also a nystagmus called *Periodic Alternating Nystagmus* [PAN], characterized by beating in one direction for a period, followed by a brief pause and then a change in direction, beating in the opposite direction. These phases alternate, with one usually longer than the other. Therefore, it is crucial to observe spontaneous nystagmus for at least a few minutes. PAN undoubtedly merits further diagnostic exploration, unless the patient reports it has been present since birth, possibly being congenital. In contrast to congenital PAN, the acquired form is typically associated with oscillopsia, an illusion of movement in the visual field due to the subject's inability to adapt to continuous changes in nystagmus direction.

Similarly, if a patient exhibits a continuous oscillation of the eyes, moving incessantly from side to side like a pendulum, it is essential to inquire whether these movements have always been present. If affirmative, the scenario is likely congenital nystagmus. However, if these movements are not present from birth, it is categorized as ‘*acquired pendular nystagmus*’, which is undoubtedly central [often associated with multiple sclerosis].

Without delving into the specifics of all other potential central nystagmus types, which are less common, it is sufficient to remember that any spontaneous nystagmus differing from horizontal or horizontal/torsional nystagmus, in terms of direction or rhythm, especially when associated with evident disconjugation of eye movement, raises concerns about a central pathology. The same caution should be applied to patients whose eyes move rapidly and uncontrollably, horizontally or in all planes, with or without intervals [‘*saccadic intrusions*’].

**References**

1. Leigh, R. J., & Zee, D. S. 2006. The neurology of eye movements, edition 4 [contemporary neurology series]. NewYork:OxfordUniversityPress
2. Bondar RL, Sharpe JA, Lewis AJ. Rebound nystagmus in olivocerebellar atrophy: a clinicopathological correlation. Ann Neurol. 1984;15:474–7
