# Supplementary material for: Consensus paper on the management of acute isolated vertigo in the emergency department
Source: Intern Emerg Med. 2024 Jul 13;19(5):1181–202. doi: 10.1007/s11739-024-03664-x (PMC11364714; doi:10.1007/s11739-024-03664-x)
Supplement: Supplementary file 11 — The clinical examination of ocular motility (DOCX 18 KB) [file 11739_2024_3664_MOESM11_ESM.docx]

**The clinical examination of ocular motility**

**Eye movements**

*Ocular motility*

Eye movements result from the coordinated activity of various systems, all working collaboratively to achieve a shared objective—stabilizing the visual image on the retina. When the head remains stationary while the external environment is in motion, compensatory eye movements essential for accurate vision are orchestrated by the visuo-oculomotor system. In routine activities, simultaneous head and environmental movements are commonplace, necessitating an interaction between the vestibulo-oculomotor system and the visuo-oculomotor system. This interaction can be either synergistic or antagonistic, depending on the specific situational demands.

**Smooth pursuit**

*Clinical aspects*
To assess this category of eye movement, simply position yourself approximately one meter in front of the patient and move a target gradually and continuously along the horizontal plane [from right to left and vice versa] and the vertical plane [from up to down and vice versa]. Instruct the patient to track the target solely with their eyes, avoiding any head movements. In individuals without impairment, smooth pursuit [SP] movements demonstrate adequate precision, reaching maximum angular speeds of 40°-60°/sec. These movements exhibit an oscillation frequency of 0.5 Hz and amplitude ranging between 10°-15°.

In clinical settings, alterations in SP can arise from either a disorder affecting the centers controlling the slow pursuit system [intrinsic alteration] or a vestibular system pathology causing nystagmus, thereby interfering with SP [extrinsic alteration].

Most intrinsic pathological alterations result in a diminished gain, defined as the ratio between input and output, within the system. The most prevalent alteration involves the substitution of SP movements with multiple saccadic movements, indicative of a reduced tracking system gain leading to a delayed eye movement compared to the target's motion. Subsequently, as the image deviates from the foveal area, the saccadic system, responsible for rapid movements, is activated. This prompts a swift compensatory movement to reposition the target within the fovea. Such impairment of smooth pursuit, unaccompanied by saccadic system anomalies, signifies brainstem dysfunction and is a characteristic feature across various pathologies [vascular, inflammatory, degenerative, tumoral].

In cases where there is concurrent impairment of the saccadic system, SP exhibits a reduced and inconsistent amplitude, often accompanied by occasional saccadic corrections. This form of alteration is commonly observed in complex ophthalmoplegia and heredoataxias.

Regarding extrinsic alterations, nystagmus can impede smooth pursuit in two scenarios:

- when nystagmus persists without inhibition from visual fixation or in the presence of a central vestibular pathology affecting the reflex arc of visuo-vestibular interaction [referred to as the arc of Ito, or retino-cerebellum-vestibulo-oculomotor reflex];

- when the nystagmus reaches an amplitude exceeding 8°-10° or an angular velocity surpassing 20°-25°/sec, due to the inability of the visuo-vestibular interaction to suppress nystagmus of this magnitude.

**The saccades**

Saccadic movements enable the subject to rapidly focus on an object within the visual field. They involve a swift angular deviation of the eyes, facilitating the quick relocation of a peripheral retinal image to the fovea or, alternatively, correcting any errors in the gaze direction concerning a desired image. Notably, during this movement, vision is temporarily "blind" as the subject does not focus on objects between the current and subsequent targets. Consequently, it is essential for this movement to be executed in the shortest time possible. Thus, there is a need to perform saccadic movements with the utmost accuracy, representing a compromise between the competing requirements of speed and precision.

*Clinical aspects*

During the bedside assessment of saccades, the examiner situates himself approximately one meter in front of the patient. The patient is instructed to focus alternately, without moving their head, on two targets—one positioned to the right and another to the left for horizontal movements, and one at the top and another at the bottom for vertical movements. In individuals without impairment, saccadic movements are executed with angular speeds of the eyes ranging around 400-450°/sec. The angular velocity of the movement is primarily associated with the amplitude of the saccade. In clinical practice, movement sequences with amplitudes of 10°-15° are predominantly employed.

Intrinsic alterations in the saccadic system hold significant clinical relevance. Saccadic hypermetria, often referred to as overshoot, is characterized by the eye surpassing the target followed by a corrective return movement to center. This phenomenon is indicative of archi-cerebellar disease and typically exhibits an ipsilateral [or prevailing] association with the side of the lesion. It is frequently observed in conditions such as cerebellar tumors and infarcts. Notably, in neoplasms affecting the ponto-cerebellar angle, ocular dysmetria often manifests as an early clinical sign in comparison to limb dysmetria.

Saccadic hypometria, also known as 'frenage' or 'undershoot,' is characterized by an eye movement at a normal angular velocity during the approach phase towards the target, followed by a deceleration in the final phase. This condition is also indicative of archi-cerebellar pathology, and manifestations of cerebellar hypometria are frequently observed in heredoataxias and complex ophthalmoplegias.

In some instances, hypermetria and saccadic hypometria coexist in a disconjugated form in both eyes. The abducted eye may exhibit a hypermetric movement, while the adducted eye concurrently demonstrates hypometria. This pattern can manifest in both directions of the saccadic movement and is pathognomonic of internuclear ophthalmoplegia when ocular convergence remains intact. Internuclear ophthalmoplegia typically arises from a lesion affecting the internuclear neurons and is nearly always associated with multiple sclerosis.

In conditions like Cogan's apraxia and progressive supranuclear palsies, the saccadic movement may be completely abolished. Supranuclear palsies often commence with an inability to execute saccadic movements on the vertical plane, with subsequent impairment extending to the horizontal plane. Notably, functional disturbances in saccades typically precede those in pursuit movements.

**The optokinetic reflex**

The optokinetic system plays a crucial role in controlling compensatory eye movements necessary for stabilizing images on the retina during movements of the surrounding environment. In this scenario, the stimulus involves the movement of the entire visual field in front of the subject, prompting a slow deviation of the eyes in the direction of the stimulus, followed by a rapid phase in the opposite direction—a response known as the optokinetic reflex [OKR].

The bedside examination of the OKR is conducted using a vertically striped tape, which is horizontally slid to assess the horizontal OKR and vertically moved to assess the vertical OKR. The tape is positioned at approximately one meter from the patient, and the stripes are slid at a rate of approximately 60°/sec. The evaluation primarily focuses on the presence or absence of the OKR, its symmetry, and its direction.

The absence of OKR may be noted in conditions such as Niemann-Pick disease, progressive supranuclear palsy, and acquired oculomotor apraxia. Additionally, its disappearance has been documented after cardiac surgery.

The OKR proves pathognomonic in two specific conditions. Firstly, in congenital nystagmus, there is an inversion of the reflex, meaning that sliding the stripes to the right evokes an OKR to the right, and vice versa. Secondly, the Parinaud syndrome is characterized by an upward OKR capable of inducing convergence and retraction nystagmus.

Conducting this test is time-efficient, and significant alterations can provide valuable indications for diagnosing possible central involvement.
